# From nasal to basal: single-cell sequencing of the bursa of Fabricius highlights the IBDV infection mechanism in chickens

**DOI:** 10.1186/s13578-021-00728-9

**Published:** 2021-12-16

**Authors:** Abid Ullah Shah, Yuchen Li, Wei Ouyang, Zhisheng Wang, Jinjiao Zuo, Song Shi, Qinghua Yu, Jian Lin, Qian Yang

**Affiliations:** 1 College of Life Sciences, Nanjing Agricultural University, Wei gang 1, Nanjing, Jiangsu 210095 People’s Republic of China; 2 College of Veterinary Medicine, Nanjing Agricultural University, Wei gang 1, Nanjing, Jiangsu 210095 People’s Republic of China; 3 Institute of Veterinary Medicine, Jiangsu Academy of Agricultural Sciences/Key Laboratory of Veterinary Biological Engineering and Technology, Ministry of Agriculture, Nanjing, 210014 People’s Republic of China; 4 Institute of Veterinary Immunology and Engineering, National Research Center of Engineering and Technology for Veterinary Biologicals, Jiangsu Academy of Agricultural Sciences, Nanjing, 210014 People’s Republic of China; 5 Shanghai OE Biotech. Co., Ltd, Shanghai, 201114 People’s Republic of China

**Keywords:** Nasal cavity, Infectious bursal disease virus, Bursa of Fabricius, Single cell RNA-sequence, RNA interference

## Abstract

**Background:**

Chickens, important food animals and model organisms, are susceptible to many RNA viruses that invade via the nasal cavity. To determine the nasal entry site of the virus and clarify why avians are susceptible to RNA viruses, infectious bursal disease virus (IBDV) was selected because it is a typical avian RNA virus that infects chickens mainly via the nasal route.

**Results:**

First, we found that IBDV infected the posterior part of the nasal cavity in chickens, which is rich in lymphoid tissue and allows the virus to be easily transferred to the blood. Via the blood circulation, IBDV infected peripheral blood mononuclear cells (PBMCs) and was transferred to the bursa of Fabricius to damage the IgM + B lymphocyte population. Subsequently, the single-cell RNA sequencing (scRNA-seq) results suggested the more detailed response of different bursal cell populations (B cells, epithelial cells, dendritic cells, and fibroblasts) to IBDV. Regarding B cells, IBDV infection greatly decreased the IgM + B cell population but increased the IgA + B cell population in the bursal follicles. In contrast to B cells, bursal epithelial cells, especially basal cells, accumulated a large number of IBDV particles. Furthermore, we found that both innate RNA sensors and interferon-stimulated genes (ISGs) were highly expressed in the IBDV-infected groups, while *dicer* and *ago2* expression was largely blocked by IBDV infection. This result suggests that *dicer*-related RNA interference (RNAi) might be an effective antiviral strategy for IBDV infection in avian.

**Conclusion:**

Our study not only comprehensively elaborates on the transmission of airborne IBDV via the intranasal route and establishes the main target cell types for productive IBDV infection but also provides sufficient evidence to explain the cellular antiviral mechanism against IBDV infection.

**Graphical Abstract:**

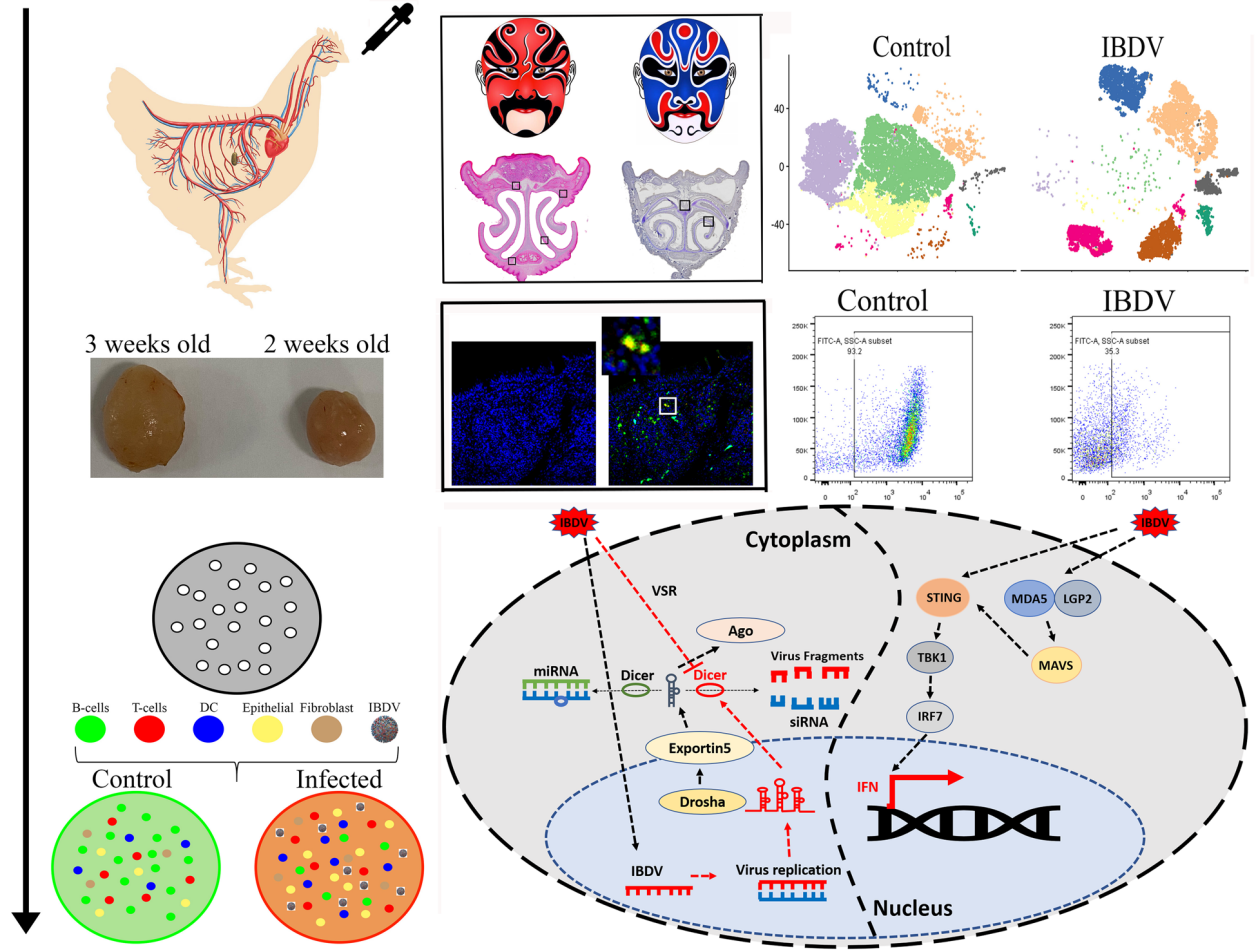

**Supplementary Information:**

The online version contains supplementary material available at 10.1186/s13578-021-00728-9.

## Introduction

It is widely accepted that aerosol or respiratory droplets dominate virus transmission in humans and livestock [1, 2]. Surveys of different poultry RNA viruses suggest that the entry of airborne particles via the mouth, nose, or eyes is the main cause of transmission [3–6]. Among these routes, viral entry via the nasal route results in immune activation due to the immune status of the nasal mucosa, which contains abundant microfold cells (M cells), dendritic cells (DCs), and T cells, which is suitable for immunoglobulin A (IgA) production to combat viral infection [7]. Thus, determining the exact entry site in the chicken nasal cavity and the infection mechanism would provide better insight into halting viral spread in the host.

Chickens are important food animals and model organisms. Chickens are also susceptible to and the source of many RNA viruses [infectious bursal disease virus (IBDV), avian influenza virus (AIV), and infectious bronchitis virus (IBV)], since all these viruses enter and infect avians via the nasal opening. Among these viruses, IBDV is an important immunosuppressive virus in chickens. The IBDV genome consists of two linear double-stranded RNA (dsRNA) segments, segment A and segment B. Segment A contains two open reading frames (ORFs). The first ORF is 3.2 kb long and encodes viral protein (VP)2 (an outer capsid protein of 47 kD), VP4 (a protease of 28 kD), and VP3 (a scaffold protein of 32 kD) [8], while the second ORF encodes VP5 (a nonstructural protein of 17 kD) [9]. Segment B is 2.9 kb long and encodes VP1 (an RNA-dependent RNA polymerase of 91 kD) [10]. This virus infects chickens via the nasal cavity and spreads to the bursa of Fabricius (BF), located in the caudal region of the chicken. Previous studies have indicated that gut-associated macrophages respond to transfer IBDV from the digestive tract to the bursa and other peripheral organs after oral infection [11]. However, how IBDV is transferred from the nasal cavity to the BF is still unclear. Hence, our study determines the detailed entry site or infected cell in the avian nasal cavity and elucidates the intranasal infection route of IBDV.

The bursa is a primary lymphoid organ in chickens and is responsible for B cell development within its microenvironment and for antibody generation following exposure to pathogens. The chicken bursa consists of a large number of follicles, which are composed of B lymphocytes, DCs, and epithelial cells. IBDV infection results in massive destruction of the bursa and the death of IgM + B lymphocytes [12], while it is still unclear how other immune and nonimmune cells in the BF respond to IBDV infection. Single-cell RNA sequencing (scRNA-seq) enables researchers to study the biological properties of an individual cell and the subsequent response of these properties to viral infection [13–15]. Recent studies have used scRNA-seq to clarify the underlying immune response to, pathogenesis of, and recovery from highly contagious viruses such as severe acute respiratory syndrome coronavirus-2 (SARS-CoV-2), influenza virus, and human immunodeficiency virus (HIV) [16–18]. Thus, via scRNA-seq, we identified avian bursal cell subsets that may sustain damage during IBDV infection and clarified the primary target cells of IBDV in the BF.

Avians are the bridge hosts closest to mammals, but their antiviral strategies are quite different. In mammals, interferon is a critical player in antiviral responses. Interferon production depends on IFN-stimulated gene (ISG) regulation, acting as a major innate immune response against viral infection. Less effort has been focused on the detailed antiviral mechanism in chickens. In addition, research on avian viruses is conducted mostly in mammalian cells [19], leading to incorrect guidance for clarifying avian virus infection. Hence, elucidating the underlying antiviral mechanism in chickens would greatly facilitate host defence against numerous and complicated avian viruses. Our study clearly shows that IBDV can easily infect both nasal and bursal cells. For RNA viruses, RNA interference (RNAi) might be a potent antiviral tool. Although some avian double-stranded RNA (dsRNA) viruses show responses to RNAi in mammals and insects, the role of RNAi in chickens remains largely undefined. The present study was conducted to evaluate the efficiency of RNAi in defending against IBDV replication.

## Results

### Intranasally inoculated IBDV infects nasal epithelial cells in chickens

**Fig. 1:**
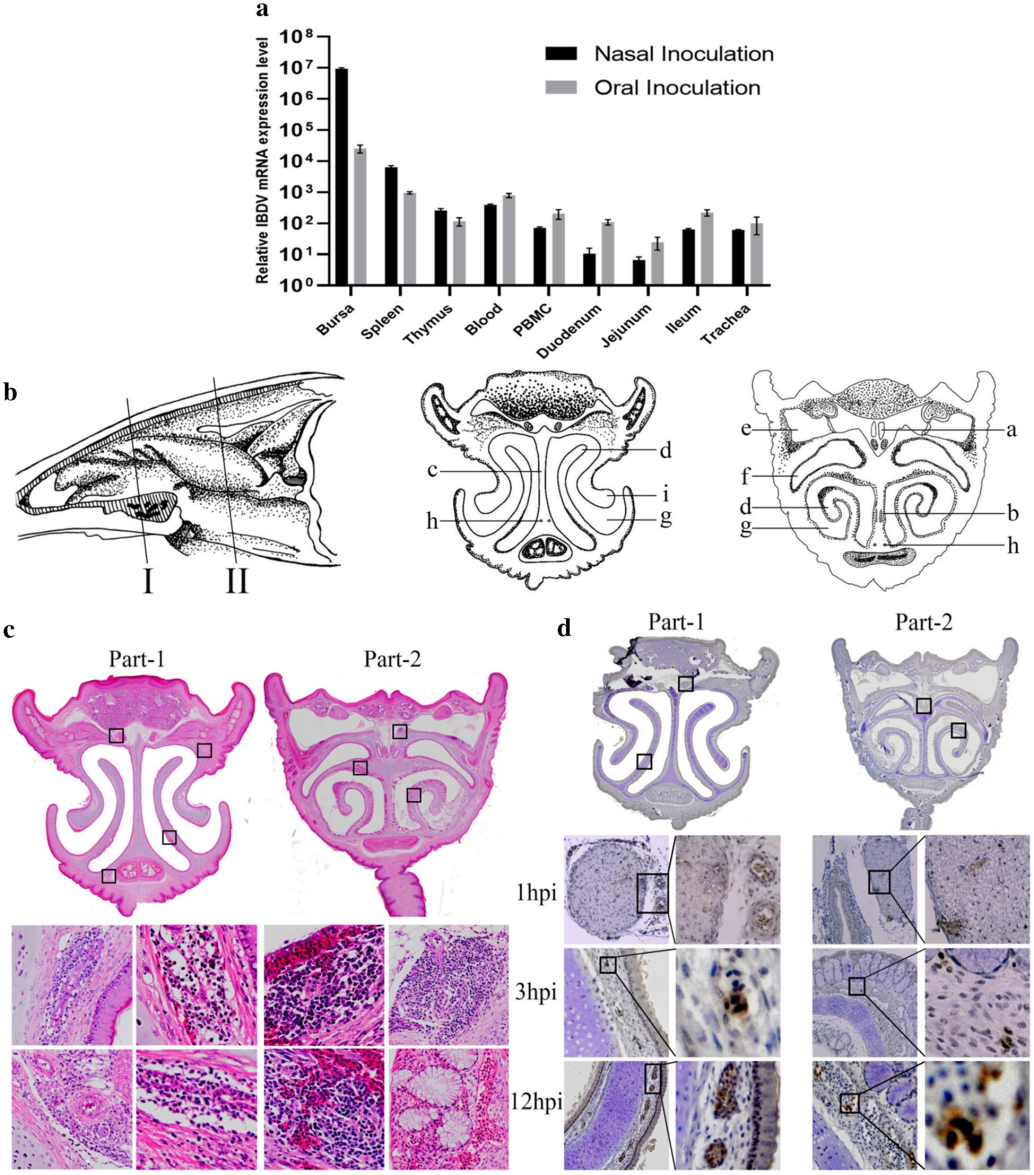
Anatomy of Nasal cavity of chicken. **a** The evaluation of IBDV’s replication efficiency with different oral or nasal inoculation via qRT-PCR IBDV mRNA expression. **b** Anatomical structure of nasal cavity from chicken through a vertical section, and a transverse sketch of two cross-sections (I and II) from chicken’s nasal cavity. (a) concha nasalis rostralis, (b) optic nerve of nervus trigeminus, (c) nasal septum, (d) concha nasalis media, (e) infraorbital sinus, (f) inferior nasal meatus, (g) nasal meatus, (h) maxillary nerve of nervi trigeminus, and (i) vertical lamella of nostril. **c** Hematoxylin & Eosin (HE) stained panoramic cross-section I and II of chicken’s nasal cavity. Diffuse lymphoid follicle and Nasal-associated lymphoid tissues (NALT) are shown in the black boxes located at concha nasalis media and distributed at the dorsal side of choanal cleft and nasal septum. **d** Immunohistochemistry (IHC) staining of cross-section I and II showing the distribution pattern of IBDV VP2 positive cells at follicle-associated epithelium (FAE) and NALT of chicken’s nasal cavity. The number of VP2 positive cells on FAE or NALT were counted at 1hpi, 3hpi, and 12hpi near to concha nasalis rostralis, on the optic nerve of nervus trigeminus and concha nasalis media. The IBDV VP2 positive cells in different parts at different times were counted in four random fields (40 ×) from three cross-sections. The scale bar represents 1 mm for whole nasal panoramic cross-sections, 40× and 100× for all other fields. All data shown are the mean results from three independent experiments

Previous studies demonstrated that aerosol or respiratory droplets containing infectious bursal disease virus (IBDV) could result in infection of chickens via the nasal and oral routes. To determine the most effective route for IBDV infection, chickens were infected with an IBDV strain (BC6/85), and samples were collected from different organs 72 h post infection (hpi). Infection via both the nasal and oral routes showed the ability of IBDV to infect the bursa of Fabricius (BF) and spleen. However, infection via nasal inoculation resulted in higher titer in the bursa than did the oral administration route (Fig. 1a). To further detect the cell tropism in the chicken nasal cavity after IBDV infection, chicks were inoculated intranasally with IBDV and divided into four groups (I. control, II. IBDV 1 hpi, III. IBDV 3 hpi, and IV. IBDV 12 hpi).

A vertical sketch of the chicken nasal cavity is shown to define the cross-section (CS) presentation (Fig. 1b). Two CSs with the same interval were then selected to show the detailed anatomical structure of the chicken nasal cavity (Fig. 1b). Next, we performed haematoxylin & eosin (HE) staining of CS-I and CS-II to identify the lymphoid tissues in the chicken nasal cavity and the differences in lymph cells at different time intervals after IBDV infection (Fig. 1c). The results showed that CS-I contained stratified squamous epithelium, while CS-II contained pseudostratified epithelium with a large amount of lymphoid tissue. Nasal-associated lymphoid tissues (NALTs) were mainly present under the epithelial wall of the inferior nasal meatus and in the lamina propria near the choanal cleft (shown in the black boxes in Fig. 1c). The follicle-associated epithelium (FAE) of NALT is located in the concha nasalis media, with few diffused lymphatic follicles, mainly in CS-II (Fig. 1c). Moreover, we performed immunohistochemical (IHC) staining of the IBDV VP2 to find the entry site of the virus in the nasal cavity. Kang previously described that the absorption capacity of the nasal vestibule of the chicken nasal cavity is very low [20]. Similarly, we observed few VP2-positive particles in CS-I. Few positive puncta were observed at the ophthalmic nerve of the trigeminal nerves and near the concha nasalis rostralis in both sections at 1 hpi. In contrast, many positive cells accumulated at 12 hpi in the posterior part of the nasal cavity, near the ophthalmic nerve and maxillary nerve of the trigeminal nerves, which might allow early virus transmission into the blood. Furthermore, we observed the absorption of VP2 into diffuse lymphatic follicles in the concha nasalis media in CS-I at 3 hpi and found that VP2 was more abundant in CS-II at 3 hpi and 12 hpi (Fig. 1d). These results indicate that IBDV infects and crosses the interior cross-section (CS-II) of the chicken nasal cavity, from which it may be transferred into the blood or be transported via the intestinal route. However, IBDV did not severely affect NALT (nasal lymphocytes).

### Replication and distribution of IBDV in chicken blood and PBMCs

**Fig. 2:**
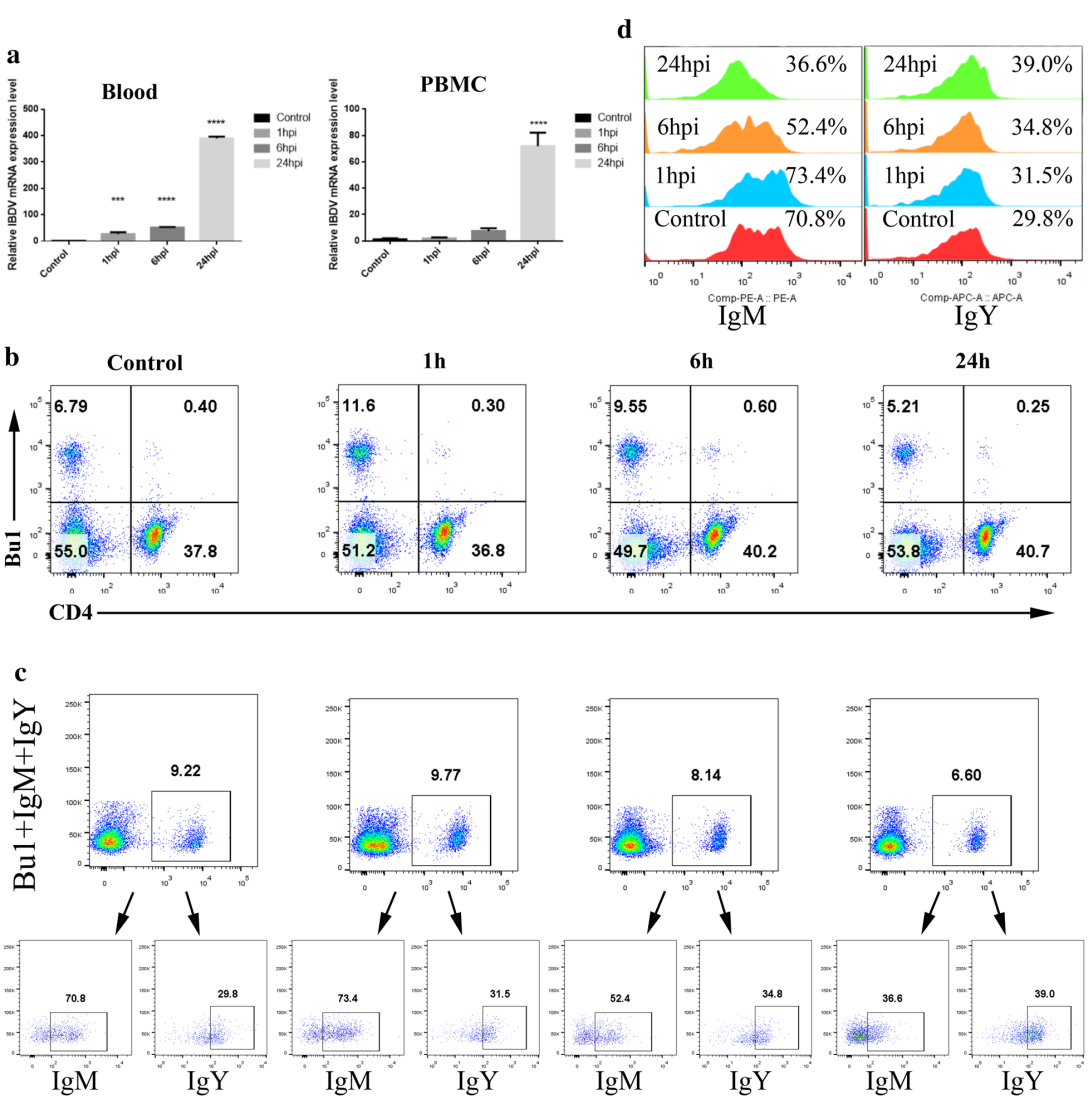
Replication and distribution of IBDV on chickens’ blood and PBMCs. **a** RNA expression levels of control and IBDV infected chicken in blood and PBMC at different time points after intranasal IBDV inoculation, n = 3 from 3 chickens per group. **b** FACS analyses of control and intranasally IBDV challenged chickens at indicated times. PBMC were isolated from each group and gated for Bu1 (marker of B cells), CD4 + T cells, and **c** and **d** immunoglobulins (IgM + and IgY +), quantification of FACS IgM + and IgY + cells are shown in the histogram at indicated time. n = 4 from 3 chickens per group. All data shown are the mean results ± SD from three independent experiments. Statistical significance was determined using one-way ANOVA. Significance difference were ***p < 0.001, ****p < 0.0001

It has been reported that following IBDV replication in the bursa at 11 hpi, the virus can enter the bloodstream to cause secondary viremia, which results in the spread of the virus to other tissues [21]. Therefore, we analysed blood and peripheral blood mononuclear cells (PBMCs) at 1 hpi, 6 hpi, and 24 hpi after intranasal inoculation of IBDV. We found that IBDV messenger RNA (mRNA) was highly accumulated at 24 hpi in both blood and PBMCs (Fig. 2a). Fluorescence-activated cell sorting (FACS) analysis showed an increase in B cells at 1 hpi and 6 hpi compared to the population in uninfected control chickens. The B cell population decreased dramatically between 6 and 24 hpi (percentage of Bu1-positive cells ranging from 9.55 to 5.21%), while little difference was observed between 1 and 6 hpi (Fig. 2b). FACS analysis also showed an increased population of CD4 + T cells between 1 and 6 hpi (ranging from 36.8 to 40.2%, Fig. 2b). Between 1 and 24 hpi, the population of IgM + B cells gradually decreased, while that of IgY + cells increased (Fig. 2c and d). In summary, the virus rapidly enters the blood (within 1 hpi), and loss of IgM + B cells occurs after 6 hpi.

### Infection and replication of IBDV in chicken bursa

**Fig. 3:**
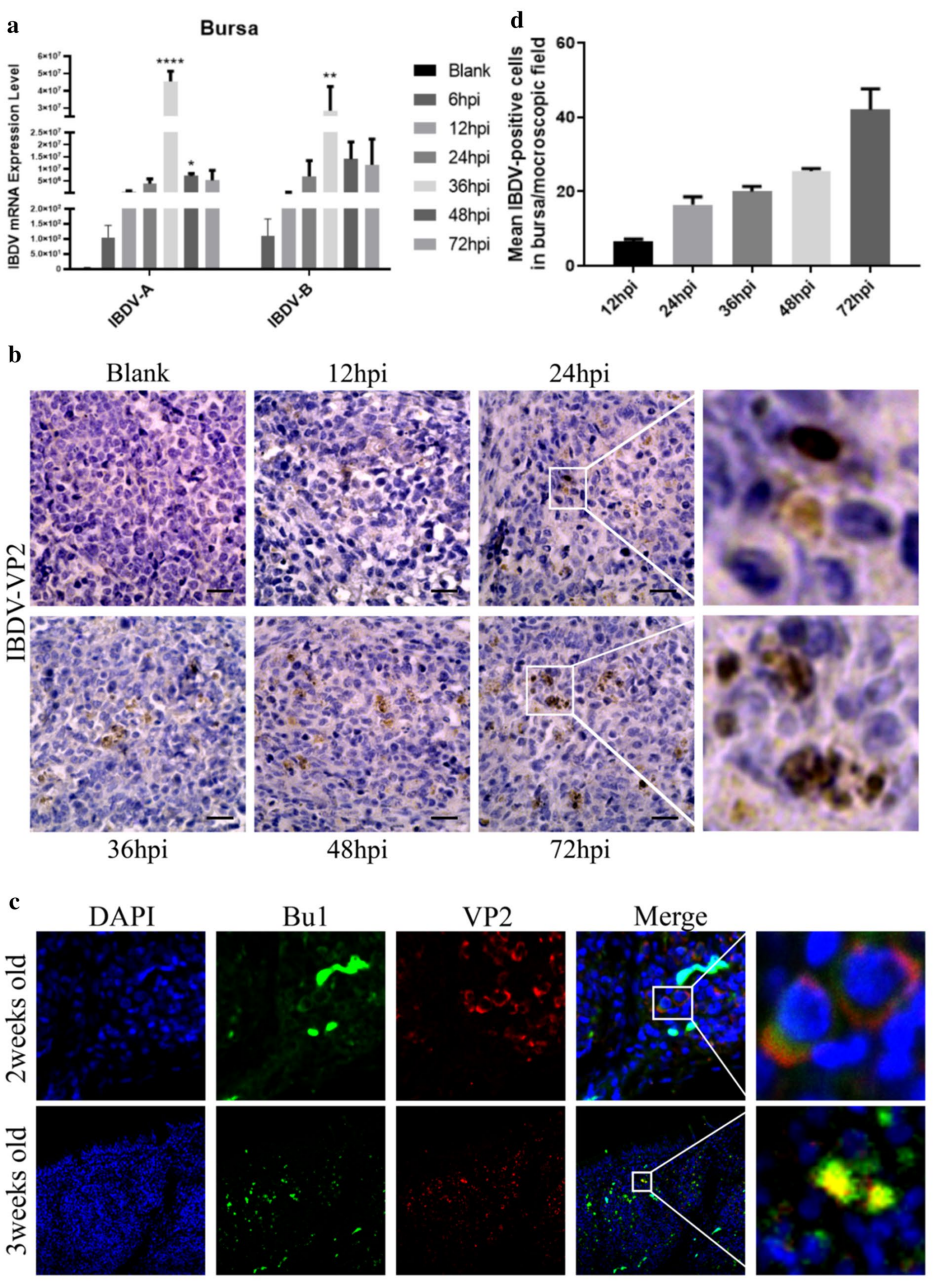
Replication and distribution of IBDV on chickens’ Bursal. **a** mRNA expression level of IBDV segments IBDV-A and IBDV-B in chickens followed by intranasal infection of IBDV strain BC6/85 shown at an indicated time interval, n = 4 from 3 chickens per group. **b** Detection of viral antigens IBDV VP2 in bursa at different time intervals. SPF chickens were inoculated intranasally with IBDV strain BC6/85. BF was harvested at 12, 24, 36, 48, and 72 h post-infection (hpi). Paraffin sections were prepared to detect the distribution of viral antigens by IHC staining using mouse anti-IBDV antiserum. Representative sections of bursa (upper left control; 40×) and infected (indicated times; 40×) were shown. **c** Two-weeks-old and three-weeks-old SPF chickens were inoculated with IBDV strain BC6/85 for co-localization of IBDV-VP2 and B cells in bursa. Bursa were harvested at 72hpi and sections of bursa (10×) were prepared to co-localize B cells with IBDV by immunofluorescence (IF) double staining. The first primary antibody was mouse anti-IBDV antibody, detecting IBDV protein VP2, and was visualized with PE conjugate-goat anti-mouse IgG. The second primary antibody was FITC mouse anti-chicken Bu1, detecting B cells. An overlay of IF pictures was shown (Merge) with an extra zoom picture (100×) of double-stained positive cells. IBDV-VP2 positive cells, Bu1 positive cells, and double-positive cells were counted in five fields/bursa/chicken at 40× magnification. **d** Brown dot distribution in IHC indicates the positive staining of IBDV VP2 positive cells counted (40×) in four fields/bursa/chicken at each designated time point. Control represents virus-free control groups. The values represent the mean ± SD from four bursa

Since the BF was the main target organ for IBDV infection, we then investigated the infection and replication of IBDV in the bursa. IBDV was administered intranasally, and samples were collected at different time points for analysis. First, IBDV was found to reach the BF as soon as 6 hpi but remained at low levels between 6 and 12 hpi. The genome of the classical IBDV strain (BC6-85) we used in this study has two segments (segments A and B). The peak IBDV level was observed after 36 hpi, accompanied by high levels of both segments (segments A and B) (Fig. 3a). Second, IHC staining showed abundant viral VP2 proteins in the bursal follicles at this time point. The number of VP2-positive cells started increasing early (12 hpi) in the bursa and peaked at 72 hpi. Viral antigens were scattered throughout the interfollicular interstitium as well as the cortex and medulla of the bursa (Fig. 3b, d). In contrast to the qPCR results, the IHC staining results indicated little expression of either IBDV segment (segment A or B) at 12 hpi, while the expression of both segments peaked at 36 hpi (Fig. 3a, b, d). These data indicate that IBDV mainly infects and replicates in the bursa in 3-week-old chicks and that VP2 accumulates at the early stage of infection. Third, HE staining showed that IBDV infection (72 hpi) led to lymphocytic necrosis, depletion of lymphoid follicles, heterophil accumulation, bursal oedema, and congestion in bursal lesions, consistent with the results of Ma [22] (Additional file 1: Fig. S1A). Since the bursa is mainly composed of B cells, we attempted to confirm whether IBDV can infect and replicate in bursal B cells. The immunofluorescence assay (IFA) results revealed some double-positive (VP2-positive and Bu1-positive) cells in both two- and 3-week-old chicks (Fig. 3c). However, numerous Bu1-positive cells in the interfollicular space of the bursa were negative for VP2 in both groups, suggesting that many of the IBDV-infected cells (VP2-positive) in the BF were not B cells. The identity of these cells requires further investigation.

### Constructing a bursa cell atlas by scRNA-seq of the host and viral transcriptomes

**Fig. 4:**
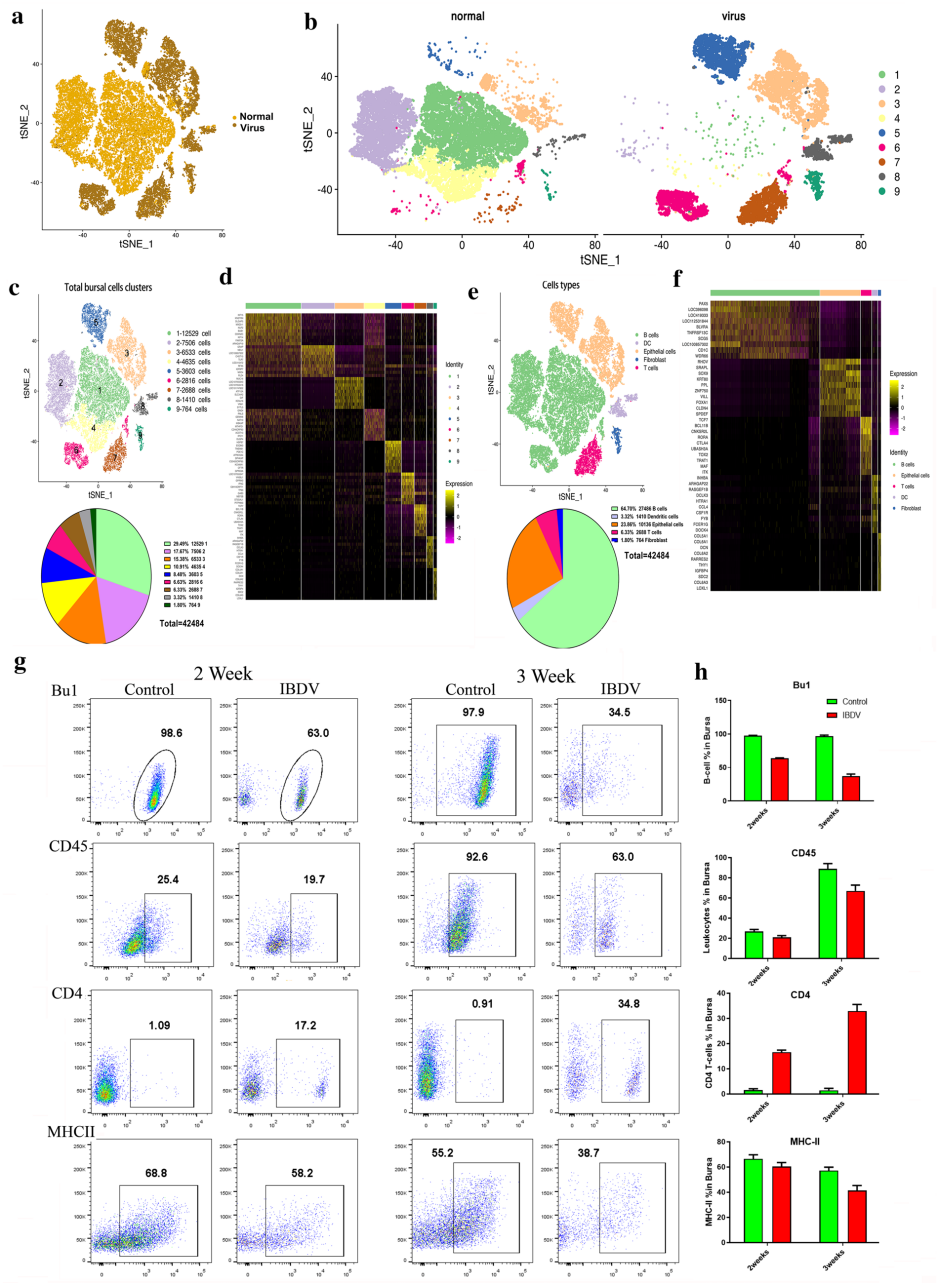
Comprehensive Bursa Map of IBDV Infection Identified by Single-Cell RNA Sequencing. **a** Aggregated data of complete bursal cells separated based on normal (light brown) and virus-infected (dark brown) as represented by t-SNE clustering. **b** t-SNE distribution of normal and virus cells into nine sub-populations colored according to the unique cell cluster and transcriptional profiling. **c** t-SNE and Pie chart Quantification of nine major cells clusters. **d** Transcriptional profiling of complete single cells 42,484 associated with nine major clusters shown in the column. Gene expressions of each specific cluster are shown for specific genes in rows. The gene expression matrix displays each clustering of cells based on different treatments (yellowish/light brown respectively for each cluster on the top of the heat map). **e** t-SNE and pie distribution of whole cell population into five different immune and non-immune cell types shown in t-SNE and unique coloured difference. **f** Heat map of transcriptional profiling of whole single cells into five major immune and non-immune cells shown in the column. Gene expressions of each cell type are shown for specific genes in rows. The gene expression matrix displays each clustering of cells based on expression level (yellowish/light brown respectively for each cell type on the top of the heat map). **g** and **h** Cells are also gated for Bu1 (B cells marker), CD45 (leukocytes and hematopoietic cell marker), CD4 + T cells, and MHCII, and the quantification for FACS are shown for each marker (green represent control and red represent infected) in each group. n = 4 from 3 chickens per group. All data shown are the mean results ± SD, and statistical significance was determined using one-way ANOVA. Significance difference was expressed as, *p < 0.05, ****p < 0.0001

In addition to B cells, we would like to know which other cells in the interfollicular space of the bursa might also be targets for IBDV infection. To answer this question, it was important to understand the IBDV infection cycle. To this end, scRNA-seq of control and IBDV-infected whole bursal cells was performed to determine how IBDV invades and destroys bursal cells. First, all bursae were divided into the control and virus-infected groups based on the samples collected from the bursae of control and IBDV-infected chickens (Fig. 4a, b). A total of 42,484 single transcriptomes were obtained and further distributed into nine different clusters based on the differential expression levels in the cell (Fig. 4c, d and Additional file 10). Each cluster was then annotated based on well-established gene markers [13]. We then divided these cells into five clusters—two clusters of nonimmune cell types (epithelial cells, 23.86%; and fibroblast cells, 1.80%) and three clusters of immune cell types (B cells, 64.70%; DCs 3.32%; and T cells, 6.33%) (Fig. 4e, f Additional file 1: Fig. S1B, and Additional file 11). Subsequent FACS analysis confirmed the presence of these populations (Fig. 4g). In addition to the B cell population, the proportion of CD45-positive cells (haematopoietic cells/leukocytes) was decreased in both the 2-week-old and 3-week-old groups, while the proportion of CD4 + T cells was increased in both groups (Fig. 4g and h).

Age may be a factor that determines the response to IBDV infection, and previous studies have reported that 3-week-old chicks are more susceptible to IBDV infection than younger chicks [21]. In this context, we found that the subclassification of cells indicated that the cell number in 3-week-old chicks was less than that in 2-week-old chicks (Additional file 2: Fig. S2A-C), consistent with our flow cytometry results (Fig. 4g and h). Based on the genomic sequence of IBDV BC6-85, both viral segments (segments A and B) were evaluated across all clusters to determine which cell subtype in the bursa is infected by IBDV. The Viral BLAST results showed that viral genome sequences were detected in Clusters 3, 5, 6, 7, and 8, while no viral genome sequences were observed in Cluster 4 throughout the cell population (Additional file 2: Fig. S2D, E). This finding drew our attention to clusters with a high proportion of viral genome sequences (Clusters 3, 6, and 8) and clusters with a complete absence of viral genome sequences (Clusters 2 and 4). As expected, no trace of any viral transcript was detected in the control groups.

### Annotation of chicken bursal B cells

**Fig. 5:**
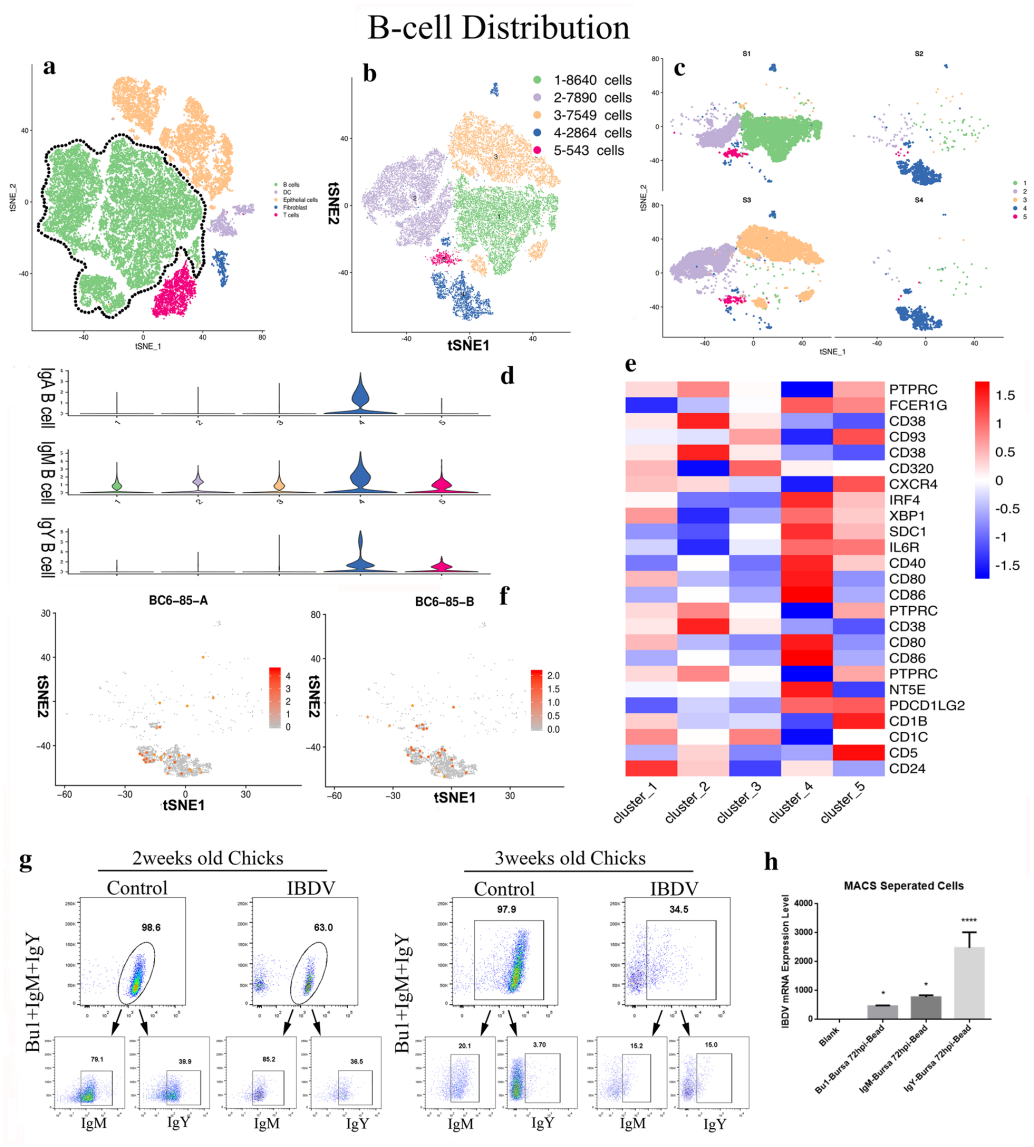
Single cells analysis of B cell distribution in bursal and FACS validation the subtype of bursary B cells change after IBDV infection. **a** t-SNE visualization of the major five cell types characterized with a different colour. The encircled green cells show the total B cell population.** b** t-SNE graph of the total B cell population divided into five clusters based on mRNA transcriptional profiling, shown with the unique colour difference.** c** Five B cell clusters distributed into normal and IBDV infected host groups shown in different tSNE graphs. S1 represents control 2-weeks-old-chick, S2 represents IBDV infected 2-weeks-old-chick, S3 represents control 3-weeks-old-chick and S4 represents IBDV infected-3-weeks-old chick. **d** Fraction of immunoglobulins of B cells (IgA, IgM and IgY) shown in different violin plots distributed into the five clusters of B cells. **e** Heatmap of the gene expression highly expressed in B cell population shown in five major cluster distribution. **f** t-SNE representation of the viral load (shown in stars) of IBDV strain BC6/85 segment A and B in IBDV infected hosts. **g** FACS analyses of bursal cells of two weeks old and three weeks old control and infected SPF chickens followed by intranasal inoculation of IBDV strain BC6/85 after 72 hpi. Bursal cells were isolated from each group and gated for Bu1 (marker of B cells), and immunoglobulins cells (IgM + and IgY +). **h** Immunoglobulins stained samples along with Bu1 were purified with MACS magnetic microbeads based on specific antibodies for viral load detection in each sample. The blank group contains the antibody-free and virus-free samples. Statistical significance was determined using one-way ANOVA. Significance difference was expressed as, *p < 0.05, ****p < 0.0001

B cells are the primary cells targeted by IBDV in the bursa and compose the largest population of cells. We first observed the influence of IBDV on the bursal B cell population. Initially, B cells in the dataset were segregated into five different clusters based on differential surface marker expression (Fig. 5a, b, e, and Additional file 12). Together, Clusters 1, 2, and 3 accounted for more than 90% (24801/27486) of the total number of B cells isolated from the control groups (Fig. 5c). However, these B cells isolated from the IBDV-infected groups were mostly segregated into Clusters 4 and 5 (Fig. 5c). Next, we examined the B cell immunoglobulin (Ig) repertoire in the bursa after IBDV infection. We differentiated the Ig subtypes (IgM, IgY, and IgA) based on marker gene expression, i.e., *LOC107050812* (IgM), *LOC107051274* (IgY), and *VH26L1* (IgA) (Fig. 5d). These Ig subtypes were overrepresented in Clusters 4 and 5, favouring the B cell repertoire and antibody recruitment only in the infected groups (Fig. 5d). The detailed results showed that IgM was expressed throughout all B cell populations. In contrast, IgY and IgA were abundant in Cluster 4, indicating that both of these Ig subtypes were secreted only by the infected bursal B cell population (Fig. 5d). Interestingly, IBDV genome sequences were only observed in Clusters 4 and 5 (Fig. 5f). Consistent with the scRNA-seq results, the flow cytometry results showed damage to the B cell population in the virus-infected groups (Fig. 5c and g). The B cell population decreased from 98.6 to 63.0% in the two-week-old group after 72 hpi, while the destruction of B cells was more severe in three-week-old chicks, with percentages ranging from 97.9 to 34.5% between the control and infected groups (Fig. 5g). Moreover, we performed flow cytometric analysis to validate the scRNA-seq data. Similarly, the flow cytometry results demonstrated that the number of IgM + and IgY + B cells was substantially decreased after IBDV infection, especially in three-week-old chicks (Fig. 5d and g). Additionally, qPCR evaluation of magnetic-activated cell sorting (MACS)-separated B cells showed high IBDV replication in IgY + B cells in the bursa (Fig. 5h). These results showed that the B cell Ig repertoire is related to the age of the chickens and that IgY and IgA play an important role in disease spread and class switching after IBDV infection. Therefore, we examined the genes responsible for class switching of IgA (*BCL6*, *PAX5*, *IRF4*, and *BLIMP1*) and found that *BLIMP1* and *IRF4* were highly expressed but *PAX5* and *BCL6* showed low expression levels in the IBDV-infected groups. This result indicated that IBDV infection accelerates IgA secretion in the bursa (Additional file 3: Fig. S3A).

### Annotation of bursal epithelial cells

**Fig. 6:**
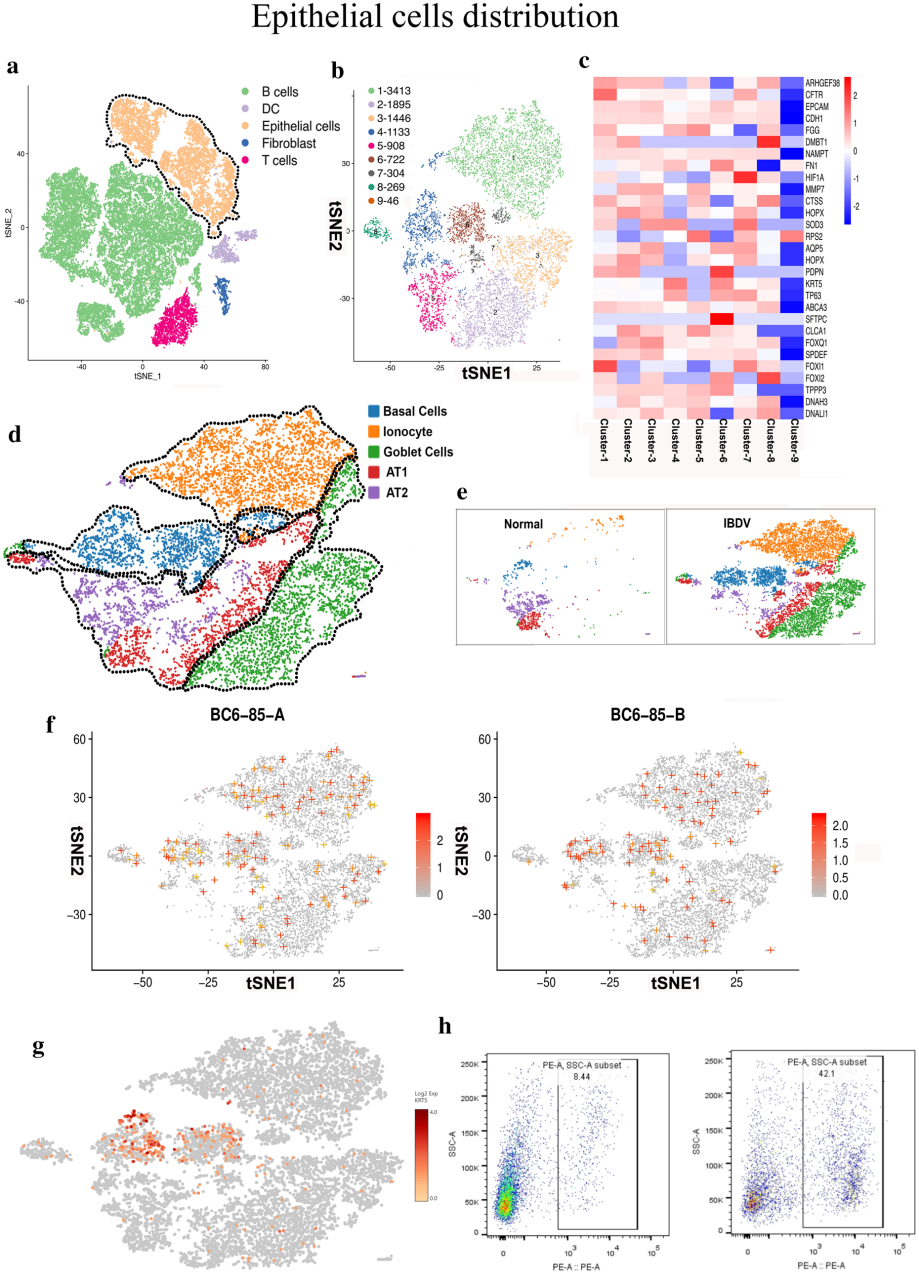
Epithelial cells distribution in bursal cell’s population and IBDV distribution in the subtype of epithelial cells. **a** t-SNE representation of the major five cell types shown in different color distribution. The encircled yellow cells showed the aggregate epithelial cells population. **b** t-SNE visualization of the entire epithelial cells population classified into nine sub-populations based on the unique mRNA transcriptional profiling, shown in different colors. **c** Heatmap of the highly expressed genes in epithelial cells population shown in nine sub-population differences.** d** t-SNE visualization of epithelial cells into four subtypes (Basal cells in blue, Ionocytes in orange, goblet cells in green, and AT1/AT2 like cells in violet color) based on differential gene expression levels shown in a different color.** e** t-SNE representation of four epithelial cells subtypes into normal and IBDV infected hosts samples defined through color distribution.** f** t-SNE graph of the IBDV strain BC6/85-A and BC6/85-B viral load (shown in colored plus) in IBDV infected hosts samples. **g** tSNE graph of the *KRT5* (shown in colored) in epithelial cells. **h** FACS analyses of *KRT5* basal cells in Control and IBDV infected chicken bursa after 72 hpi. Bursal cells were isolated from each group and gated for *KRT5* (marker of basal cells)

The epithelial cell population contained 10,136 cells, which accounted for 23.86% of the total cell population (Fig. 6a). First, the epithelial cells were divided into nine subpopulations based on their gene expression patterns. Further identification based on highly expressed surface markers resulted in organization of this population into five different subepithelial cell types (ionocytes, goblet cells, basal cells, AT1 cells and AT2 cells) (Fig. 6b, c, and d, Additional file 13). Second, since we did not detect high accumulation of IBDV in the largest population of B cells (Figs. 3c and 5f), we then evaluated whether bursal epithelial cells (the second largest population) may be able to accumulate IBDV genome sequences. The results showed that both segments (segments A and B) were present and accumulated in bursal epithelial cells. Further analysis found that most IBDV genome sequences had the greated accumulation in bursal basal cells, while a small part of IBDV genome sequences had beed found in bursal ionocytes and goblet cells, albeit at a low level (Fig. 6e, f). Basal cells were characterized by high expression of cytokeratin-5 *(KRT5)* and tumor suppressor protein p63 (*TP63)*; these genes were enriched in the infected groups, mostly in Clusters 4 and 6 of epithelial cells (Fig. 6f).

Multipotent basal cells are mainly identified by their expression of *KRT5* and *TP63* [23–25]. These basal cells are reported to play an important role in maintaining the homeostasis of the airway epithelial surface because of their unique ability to self-renew and differentiate into epithelial cells of various lineages [24]. Therefore, we found that most *KRT5* was localized in the basal region in Clusters 4 and 6 of epithelial cells (Fig. 6g). Interestingly, the *KRT5*-expressing cells and IBDV genome sequences were distributed throughout the epithelial cell population in the same manner (Fig. 6f and g). Third, the flow cytometry results showed that *KRT5* replicates after IBDV infection in bursal epithelial cells (Fig. 6h). Together, these results indicate that IBDV is most prevalent in the basal cells of the bursal epithelium in addition to the B cell population, and further, that *KRT5* facilitates viral replication and distribution throughout the bursa.

### Annotation of bursal dendritic cells

The above results demonstrated that basal cells are the main target cell for IBDV infection, and we then sought to determine which cells take up IBDV and migrate into and out of the bursa. The cortical region of the chicken bursa contains mesenchymal cells and macrophages, whereas mostly lymphocytes, epithelial cells and dendritic cells are present in the medullary region [26]. Therefore, we evaluated whether bursal DCs and macrophages are the transport cells of IBDV. First, we identified and divided the total DC population (1410, 3.32%) into four distinct clusters (Additional file 4: Fig. S4a, b) based on their surface markers. We found that Clusters 1 and 3 mostly consisted of macrophages (M1 and M2), while Clusters 2 and 4 contained large populations of DCs (cDCs, pDCs and MoDCs) (Additional file 4: Fig. S4b, c, e and Additional file 14). Moreover, we found that 90.57% of the DCs were present in the infected groups, constituting 62.97% of the cells in the 2-week-old infected group and 27.58% of cells in the 3-week-old infected group (Additional file 4: Fig. S4d). Furthermore, we found that vmRNA of IBDV was distributed throughout the infected groups and was especially prevalent in macrophage clusters compared to the DC clusters, while no trace of vmRNA was found in the control group (Additional file 4: Fig. S4f). Finally, chickens contain some unique DC types, such as bursal secretory dendritic cells (BSDCs) in the epithelial environment of the bursa, follicular dendritic cells (FDCs) in the germinal centre, and Langerhans dendritic cells (LDCs) located in the skin epithelium [27]. Hence, we grouped BSDCs and FDCs based on the expression of *vimentin* and *desmin*, respectively [28] (Additional file 5: Fig. S5b, c). Along with *vimentin*, BSDCs also express *DEC205* and *TRKB*, making BSDCs the most abundant cells among the DC population, accounting for 767 cells (54.32% of the total DC population). However, only 102 FDCs (7.32%) highly expressed the surface markers *C4*, *CR1L* and *desmin* (Additional file 5: Fig. S5).

#### The IFN response to IBDV infection in chickens

**Fig. 7:**
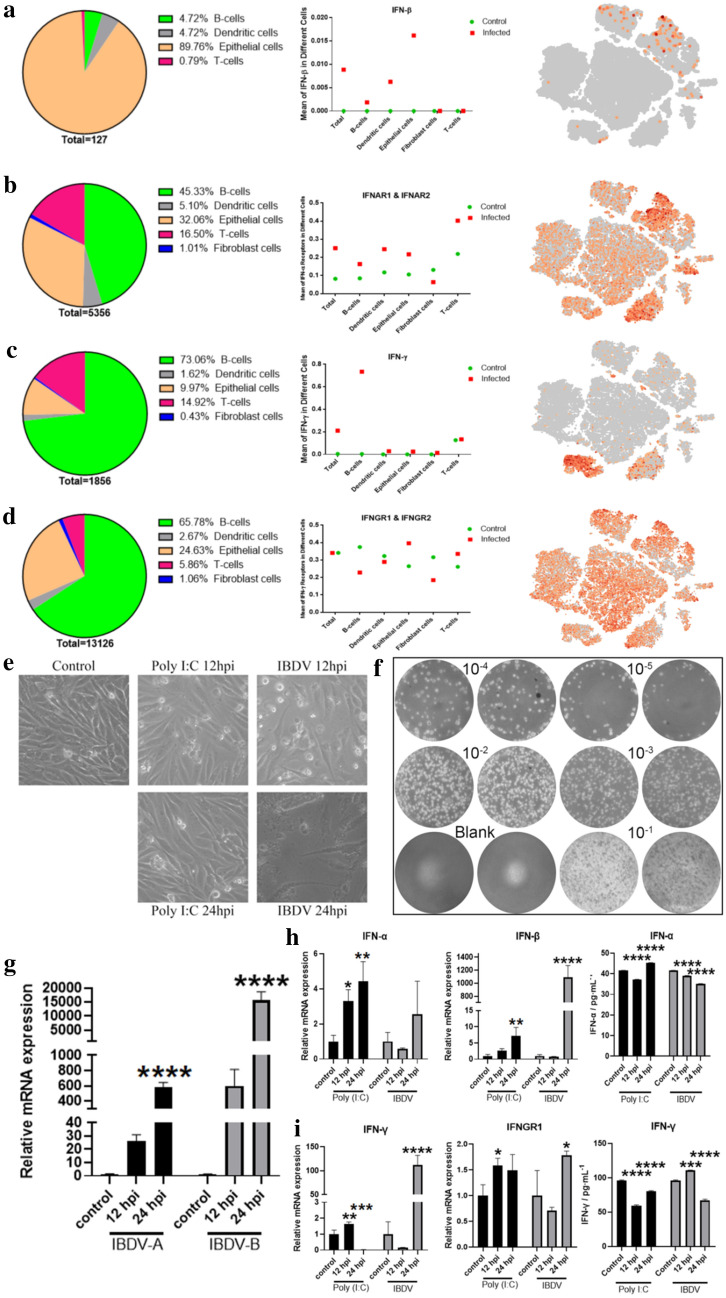
The expression of chicken interferon pathways after IBDV infection. **a** Pie chart showing the percentage of gene transcriptome level in designated cell type, graph showing the gene transcriptome level in control and infected groups in each cell type, and t-SNE distribution of gene transcriptome level in throughout the bursal cells population; of *IFN-β*; **b** *IFNAR1* & *IFNAR2* (type 1 IFN surface receptors); **c** *IFN-γ* and; **d** *IFNGR1* & *IFNGR2* (IFN-γ surface rectors). **e** Morphological observation of DF1 cells stimulated by Poly I:C and IBDV at 12hpi and 24hpi through the light microscope. **f** Plaque appearance of DF1 cells infected with the IBDV. Each group is shown in two different wells. Blank: mock cells; 10^–1^ to 10^–5^ show plaque formation by a tenfold dilution of virus stock. **g** Viral RNA expression level of IBDV segments (IBDV-A and IBDV-B) on DF1 cells followed by IBDV infection at the indicated time. **h** Relative mRNA expression level at DF1 cells after treatment with poly I:C and IBDV at 12hpi and 24hpi of *IFN-α* & *IFN-β*, and protein expression through ELISA of *IFN-α* (right side). **i** Relative mRNA expression level at DF1 cells after treatment with poly I:C and IBDV at 12hpi and 24hpi of *IFN-γ* & *IFNGR1* and protein expression through ELISA of *IFN-γ* (right side).**j** IBDV-A and IBDV-B mRNA expression level through qRT-PCR after post-treatment on DF1 cells with synthetic *IFN-γ* followed by IBDV infection for the indicated time. All the experiments are the means ± standard error from three independent repeats. All the qRT-PCR expressions were normalized with *GAPDH* mRNA expression level. The level of significance between blank and treated groups are identified by *p < 0.05, **p < 0.01, ***p < 0.001, and ****p < 0.0001, determined by one-way ANOVA with Tukey’s multiple comparison test

As an essential antiviral factor and player in innate immunity, type I interferons (IFN-α and IFN-β) in avians play a pivotal role against viral infection [29, 30]. Type II IFN (IFN-γ) serves as a bridge between innate and adaptive immunity in both avians and mammals. Therefore, we examined the IFN response after IBDV infection both in vitro and in vivo. On the one hand, the in vivo scRNA-seq results showed that IFN-β and its receptors (IFNAR1 and IFNAR2) were highly expressed in the IBDV-infected groups (Fig. 7a and b). Importantly, approximately 90% of IFN-β was found in the IBDV-infected epithelial cell population (Fig. 7a). In contrast, IFN-γ was highly expressed in only the B cell population in IBDV-infected groups (Fig. 7c). However, IFN-γ receptors (*IFNGR1* and *IFNGR2*) were expressed throughout the cell population in the control groups and also exhibited higher expression in epithelial and T cell population of the infected groups (Fig. 7d). Interestingly, our scRNA-seq results found that IFN-β expression increased with time and that IBDV accumulated with the increase in IFN-β expression in the infected groups. This result indicated that the type I IFN pathway is not a sufficient antiviral defence pathway against IBDV infection in chickens.

Combining the result that IBDV accumulated in bursal epithelial cells with the knowledge that chickens lack *RIG-1*, IFN regulatory factor 3 (*IRF3*), and *IRF9*, which are essential for activation of type I IFN production in mammals [31, 32], we then hypothesized that avian IFN has a function in defending against viral replication. To validate this hypothesis, we infected chicken embryonic fibroblast cells (DF1) with 1 µl of 1000 TCID_50_ virus/mL or poly(I:C) at the same concentration. Our results showed an early decline in cells at 12 hpi compared with that in the poly(I:C) and control groups, while complete cell damage was observed at 24 hpi (Fig. 7e). Moreover, a plaque assay showed that the viral titre became discernible and countable with each dilution, while the qPCR results showed that IBDV transcripts peaked at 24 hpi (Fig. 7f, g). Furthermore, we found that poly(I:C) stimulation enhanced the expression of both IFN-α and IFN-β, while IBDV infection decreased IFN-α expression at the protein level (Fig. 7h). Regarding IFN-γ (type II IFN) and its receptor *IFNGR1*, our study found that IBDV infection enhances their mRNA expression but decreases their protein levels (Fig. 7i). This result suggests that IBDV could regulate and block the IFN system in avian cells.

### *Dicer* plays an important role in the antiviral defence mechanism against IBDV infection

**Fig. 8:**
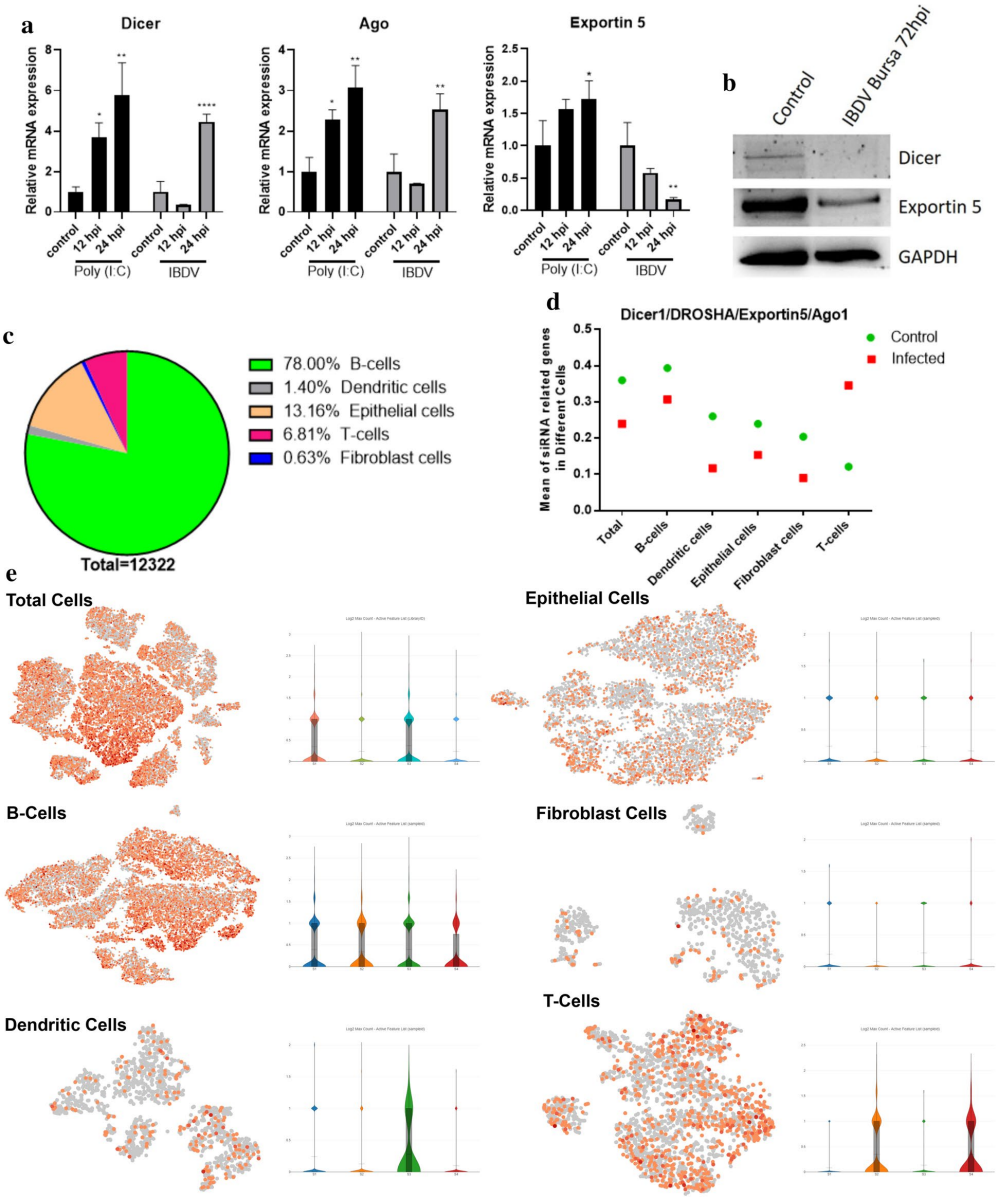
The expression of chicken *Dicer/Ago/Exportin5* pathways after IBDV infection. **a** Results of qRT-PCR analysis of *Dicer*, *Ago*, and *Exportin5*. **b** Protein expression determined by Western blot of *Dicer*, *Exportin5*, and *GAPDH*. **c** Pie chart showing the percentage of *Dicer/Drosha/Exportin5/Ago* transcriptome level in each cell type. **d** Graph figure showing the gene transcriptome level of *Dicer/Drosha/Exportin5/Ago* in control and infected groups in each cell type. **e** t-SNE (left side) and violin plot (right side) of *Dicer/Drosha/Exportin5/Ago* in each cell population. Violin plots show expression in each (control and infected) sample type. The data shown are the means ± standard error. All these expressions were normalized with GAPDH mRNA expression level. These results are taken from three independent experiments. The significance level between blank and treated groups is identified by *p < 0.05, **p < 0.01, and ****p < 0.0001, determined by one-way ANOVA with Tukey’s multiple comparison test

In addition to the IFN system, RNAi is an effective antiviral mechanism recognized by the host enzyme *dicer*, which cleaves double-stranded RNA viral genomes into siRNAs and inhibits viral replication. Mammals express DICER1 and AGO1 for the biogenesis of both miRNAs and siRNAs but retain the cleavage activity essential for RNA interference (RNAi) [33–35]. Chickens have both IFN and RNAi systems, and the balance between the functions of IFN and RNAi in chickens still needs clarification. The above study demonstrated that the avian IFN system does not defend against IBDV infection and replication. Therefore, we sought to determine whether *dicer* activates the RNAi mechanism—alone or together with *Ago* and *XPO5*—to defend against IBDV infection or only functions in microRNA biogenesis. First, we found that the mRNA expression levels of both *dicer* and *ago* were high at 24 hpi, while *XPO5* was downregulated after IBDV infection in DF1 cells (Fig. 8a). Second, the western blot results showed that Dicer protein expression was abolished after infection, while a decreased level of XPO5 was observed (Fig. 8b). These results indicate that IBDV influences miRNA biogenesis in DF1 cells after infection. Third, to assess the role of Dicer in antiviral defence mechanisms after IBDV infection, we performed scRNA-seq in the chicken bursa. We explored the expression of *Dicer*, *Drosha*, *XPO5*, and *Ago1* in the whole population of cells in the chicken bursa. The results showed that most of these genes were expressed in the B cell population (Fig. 8c). Consistent with western blot analysis, scRNA-seq showed that *dicer* and its related genes were highly expressed in the control group in all cells except for the T cell population (Fig. 8d and e). These results show that *dicer* might cleave the IBDV genome and help in an antiviral defence mechanism.

### Activation of the RNA sensing pathway after cellular recognition of IBDV

In normal chicken cells, RNA viruses are recognized by *TLR3* [36], which further promotes the activation of *MDA5* and *LGP2* or the alternative *STING/TBK1/IRF7* pathway. Therefore, to better understand the correct cellular mechanism after IBDV infection, we performed in vitro experiments and then compared the data with our in vivo scRNA-seq data. First, the qPCR results showed that all the RNA sensing pathway genes, i.e., *TLR3*, *MDA5*, and *LGP2,* were highly expressed after IBDV infection (Additional file 6: Fig. S6a). Similarly, *STING* and *IRF7* also showed high mRNA expression levels in DF1 cells (Additional file 7: Fig. S7a). Consistent with these in vitro qPCR results, the in vivo scRNA-seq data also showed high transcriptomic expression of the *TLR3/MDA5/LGP2/MAVS* RNA sensing pathway and the alternative *STING/TBK1/IRF7* pathway in the whole cell population in the infected groups (Additional file 6: Fig. S6a and Additional file 7: Fig. S7c). This result indicates that IBDV successfully activates these cellular RNA sensing pathways. Interestingly, components of these pathways were highly expressed in the epithelial cell population in the chicken bursa (Additional file 6: Fig. S6b and Additional file 7: Fig. S7b). Collectively, these results suggest that bursal epithelial cells play a very important role in IBDV replication via *KRT5*, which further activates RNA sensing pathways and finally promotes *IFN-β* expression in bursal epithelial cells.

## Discussion

Although the chicken nasal cavity has a strong and effective immune barrier system that is difficult for pathogens to penetrate, pathogens are still more likely to enter and infect the nasal cavity than other sites, predominantly via the aerosol or respiratory droplet route. Infectious bursal disease virus (IBDV) deserves more attention as a good research focus for three reasons. First, IBDV mainly invades chickens via the nasal or oral opening, which makes it suitable for studying the viral invasion pathway. Second, IBDV infection can greatly damage the population of B cells, especially IgM + B cells, which can be useful for B cell-related studies. Third, as a typical avian RNA virus, IBDV mainly causes immunosuppressive disease in chickens, making it suitable for mechanistic studies of avian virus defence systems.

In this study, we attempted to understand the mechanism of IBDV invasion via the nasal cavity. Our study was the first to find and demonstrate that IBDV infects the nasal epithelium, and more virus was observed in the posterior (lymphoid-rich) part of chicken nasal-associated lymphoid tissue (NALT) and avian olfactory cells. This pattern suggests that IBDV infects the interior (cross-section II) of the chicken nasal cavity, from which it may be transferred into the blood or be transported via the intestinal route. It has been reported that oral vaccination with IBDV leads to high levels of viral infection and replication in many organs, such as the spleen, thymus, cecum tonsils at an early stage of infection, while it also could be detected small amount in liver and kidneys [37, 38]. Therefore, we speculated that there is a possibility for airborne IBDV particles to enter via the nasal route and be transferred via submucosal DCs or high endothelial venules (HEVs) in the nasal epithelium into the blood [39]. IBDV virus was observed at the early time point of 1 hpi in the blood after nasal infection. In addition, the study also found that the total B cell population, including the IgM + and IgY + B cell subpopulations, was substantially decreased at the early stage of infection, indicating the influence of IBDV on blood peripheral blood mononuclear cells (PBMCs). Together, the results of our study suggest that airborne IBDV particles can be transferred via the nasal route, where they infect NALT and can be transferred into the blood. However, the detailed molecular mechanism of IBDV transfer from the nasal cavity to the blood is still undefined.

Previous studies demonstrated that the bursa and B cells are the main target organ and cell type for IBDV infection. We then tried to clarify how IBDV infects and influences the bursal B cell population. Chickens aged 1 to 14 days are comparatively less susceptible to IBDV, while more severe clinical symptoms are found in chicks aged 3–6 weeks [21, 40]. We thus selected 2- and 3-week-old chickens as experimental subjects. As shown in Fig. 3, IBDV was highly colocalized with B lymphocytes in 3-week-old chicks compared with 2-week-old chicks. In addition, we observed massive destruction of B cells in the IBDV-infected groups. However, there is an obvious lack of knowledge regarding whether IBDV infects macrophages and B lymphocytes, what happens to other immune cells (dendritic cells (DCs) and T cells) and immunoglobulins (Ig) after infection [11, 12, 22], and what drives the immune system of chickens to produce antibodies against this virus. Via single-cell RNA sequencing (scRNA-seq), we explored the molecular mechanism of the B cell repertoire and immune response in the chicken bursa after IBDV infection. Collectively, our findings identified five cell clusters (B cells, epithelial cells, T cells, DCs, and fibroblast cells) in the chicken BF. First, we confirmed that IBDV infection ultimately damaged the B lymphocyte population in the bursa, consistent with our flow cytometry results. Among these B cells, IgY + and IgA + cells were more abundant than IgM + cells in the B cell population in the infected groups. This result indicates that IgY + and IgA + cells might be responsible for the production of antibodies against IBDV. Consistent with the increased IgA + cell population in the IBDV-infected groups, *BLIMP1* and *IRF4* were highly expressed in the IBDV-infected groups, while *PAX5* and *BCL6* were expressed at low levels [41], which indicated increased secretion of IgA in the chicken bursa after IBDV infection. In addition, we found that *C-X-C* motif chemokine ligand (*CXCL*)*12* and *CXCL13* were highly expressed in the BF of IBDV-infected chickens, while stromal-derived factor 1 (*CXCL12*) was completely absent in the normal groups. Evidence from diverse studies shows that the *C-X-C* motif chemokine receptor (*CXCR*)*4/CXCL12* interaction may be responsible for recruiting immature B lymphocytes into the bursal mesenchyme from blood granulocytes. Then, the *CXCR5/CXCL13* interaction may induce the migration of B cells into bursal follicles (Additional file 3: Fig. S3B) [42–45]. Hence, this result shows that IBDV infection leads to B cell migration, which might be of great interest and may lead to more effective therapeutic interventions. Finally and interestingly, we only observed a lower viral genome level in the IBDV-infected bursal B cell population. One possible reason is that the damage was greatest to the B cell population after infection. If the IBDV genome was not present in enormous amounts in the bursal B cell population, it would not be unorthodox to consider that the virus may target epithelial cells.

Our scRNA-seq data found that epithelial cells were the second largest population identified in the bursa, especially in the IBDV-infected groups. Recent studies demonstrated that epithelial cells are a major source of viral spread, for example, for influenza virus in humans and mice [17, 46–48] and astrovirus in the villi of humans and turkeys [49, 50]. However, there is no evidence of viral invasion in the bursal epithelium. Therefore, our data are the first to confirm these findings and extend this result to other nonimmune cells in the chicken bursa. We found a high viral load in the basal cells clustered by expression of the markers *KRT5* and *P63*. Basal cells have been reported to be the main endogenous stem cell population in the mouse trachea and human lungs [24]. Zuo et al. found that expression of *P63* and *KRT5* promotes the proliferation and differentiation of stem cells into bronchiolar secretory cells [51]. Recently, studies identified an isoform of *Dicer*, named antiviral *Dicer* (*aviD*), that protects tissue stem cells against RNA viruses, including Zika virus and severe acute respiratory syndrome-coronavirus-2 (SARS-CoV-2), by cleaving viral double-stranded RNA (dsRNA) to orchestrate antiviral RNAi [52]. In agreement with this finding, we speculate that proliferative expansion can be induced in response to IBDV damage to bursal basal cells, which may also promote viral replication in this region.

In mammals, the innate immune response activates interferon expression, protecting differentiated cells but not stem cells against viral replication [53]. A recent study showed the molecular regulation of antiviral RNAi in mammalian stem cells [52]. *Dicer*, *Drosha*, *Exportin5* and *Ago2* play an essential role in the antiviral RNAi response in insects [54] and plants [55]. *Drosha* was reported to directly target and bind to viral RNA secondary structures, which then blocks the function of RNA polymerase to suppress virus replication. In contrast, *Dicer* directly cleaves virus-derived dsRNA to generate siRNAs, subsequently inducing the decay of viral RNA molecules [56]. Whether IFN and RNAi contribute in these ways to antiviral immunity in avians remains controversial. In our study, in contrast to the high expression of IFN, ISGs, and innate immunity-related genes in the IBDV-infected groups, our in vitro and in vivo studies elucidated that IBDV accumulation blocks dicer activity and *exportin5* in DF1 cells and the chicken BF. In addition to its inhibition in IBDV-infected cells, Dicer was also found to be quickly inhibited compared with *actin* or *Ago2* in an influenza A virus-infected Vero cell line, suggesting that dicer might be targeted and influenced by influenza A virus [57]. Multiple key questions related to this complex regulation have yet to be answered, including whether avian stem cells contain truncated dicer protein to defend against avian RNA viruses, and whether dicer only performs a role in miRNA biogenesis in chickens or also function in antiviral RNAi? There is an obvious lack of knowledge, and further research is required to address these long-standing issues.

## Conclusion

The nasal cavity is the main entry site for infectious pathogens, and the dominant routes of viral infection are aerosols and respiratory droplets. This study first demonstrated that IBDV infects chickens via the interior region of the nasal cavity and is then transferred into the blood. We then observed that IBDV infects the bursa and largely destroyed the B lymphocyte population. Secondly, we identified five main cell clusters (B cells, T cells, dendritic cells, epithelial cells and fibroblast cells) in the bursa. Among these clusters, the B cell—especially the IgM + B cell—population was severely damaged after infection, while the population of IgA + B cells, which might be responsible for the defence against IBDV, was greatly increased after infection. Third, we found that basal cells in the bursa are the primary target cells for IBDV infection and replication. Finally, our study also found that chicken IFN-related genes and ISGs were both activated by IBDV infection, while the virus still accumulated in bursal basal cells. In addition, we found that the expression of *Dicer* and its related enzymes *Ago2* and *Exportin5* was greatly blocked by IBDV replication, suggesting that RNAi, especially via *Dicer*, might be a new system for avian defence against avian RNA viruses. Taken together, our study demonstrated the airborne transmission of IBDV and IBDV transmission via the intranasal route into the BF. On the other hand, our study clearly showed that IBDV blocks dicer in vitro and in vivo, suggesting that RNAi might be a potent, effective tool in the avian antiviral defence mechanism.

## Materials and methods

### Animals, cell lines and viruses

The Specific-Pathogen-Free (SPF) white leghorn chickens were obtained from Jiangsu Academy of Agricultural Science (JAAS). Each experimental group of chickens are housed in separate high-security isolation units. A clean and ad libitum diet was provided throughout the experimental period. DF1cells were obtained from the ATCC and cultured in Dulbecco Modified Eagle Medium (DMEM) supplemented with 10% fetal bovine serum and incubated in a 5% CO_2_ incubator at 37 °C. A classical virulent IBDV strain (BC6/85) of serotype 1 was kindly provided by Dr. Ouyang wei from JAAS, Nanjing, China. Each chick in a different experimental group was inoculated intra nasally with 1000 EID_50_ (50% egg infective doses) of IBDV strain BC6/85 or 0.01 M phosphate-buffered saline (PBS) (0.1 mL per chicken). The incubation period was kept 72 h post-infection (hpi) or as otherwise stated, independent of infected or control group. All experimental procedures were conducted under the approval and supervision of the Institutional Animal Care and Ethical Committee of Nanjing Agricultural University (SYXK-2017-0007), and the National Institutes of Health guidelines were followed to perform the animal experiments.

### Experimental design and sample collection

A total of sixty 2-week-old and 3-week-old SPF chickens were randomly distributed into the infected group and control group. Chickens from infected groups were intranasally inoculated with IBDV strain BC6/85, while the control group were inoculated with PBS. According to the different experimental requirements, chickens from each infected and control group were sacrificed at 1, 3, 6, 12, 24, 48 and 72 hpi. The nasal, bursa of fabricius (BF) and blood samples from each infected and control chicken were collected from the sacrificed chicks. The BF and blood samples were collected for qPCR and flow cytometry analysis. In addition, nasal and BF were examined for histopathology by hematoxylin and eosin (HE) staining and for detecting viral antigens by immunohistochemistry staining (IHC). The infected and control groups’ BF was collected to co-localize viral antigens with B cells by immunofluorescence assay (IFA) double staining.

### Reagents and antibodies

For cell isolation from bursa, RPMI 1640 and fetal bovine serum were bought from GIBCO (Shanghai, China). ACK lysis buffer used for RBC lysis was also purchased from GIBCO. For PBMC isolation from chicken blood, Histopaque-1077 with a density of 1.077 g/mL was purchased from Sigma-Aldrich (St. Louis, USA). For magnetic beads, cell separation with specific antibody, Anti-FITC Micro-Beads, Anti-APC Micro-Beads, anti-PE Micro-Beads, MACS column, MACS assembly and MiniMACS Starting kits were all purchased from Miltenyi Biotec (Auburn, USA). Antibodies for flow cytometry and immunofluorescence assay (IFA), such as mouse anti-chicken Bu1-FITC, mouse anti-chicken CD4-PE, mouse anti-chicken CD45-APC, mouse anti-chicken MHCII-PE and immunoglobulins antibodies mouse anti-chicken IgM-PE, and mouse anti-chicken IgY-APC, were all purchased from Southern Biotech (Birmingham, AL, USA). The primary antibody used for IBDV in IHC and IFA mouse anti-VP2 polyclonal antibody was kindly provided by Dr. Ouyang wei from JAAS, Nanjing, China. Secondary antibodies for immunofluorescence analysis, such as PE-conjugated goat anti-mouse IgG (H + L), were purchased from Multi Sciences (Hangzhou, China). For cellular nucleus staining, Hoechst 33342 (1:2000, H3570) was purchased from Life Technologies (Shanghai, China). IHC detection for HRP conjugated anti-mouse/anti-rabbit IgG SABC kit was purchased from BOSTER (Wuhan, China). All other reagents and chemicals, unless otherwise stated, were obtained from Sigma. All the antibodies, reagents, chemicals, peptides, recombinant proteins, vectors, inhibitors, commercial assay kits, and software used in this study are listed in Additional file 8: Table S1 (Additional file 9: Table S2, Additional files 10, 11, 12, 13, 14).

### Histopathological staining

A total of twenty infected and six control chickens were euthanized by intravenous injection of pentobarbital, and nasal and BF tissues were collected. The nasal sample was collected from control and infected 2 weeks old chicks at 1, 3 and 12 hpi. The head was cut off, and the beak in front of the nostrils was removed, then the skin and cheek muscles were taken off. The remaining nose containing the nasal turbinates, septum, lateral walls and maxilla, was fixed in Bouin’s fluid for 72 h at room temperature [58]. After fixation, nasal blocks for histological analysis were cut according to the fractions 1/3 and 2/3, using the landmarks provided in the diagram Fig. 1b. The blocks were decalcified in 4%-paraformaldehyde and 5% formic acid in PBS for 3 days at room temperature. The BF from IBDV-infected and control chickens were collected at 12, 24, 36, 48 and 72 hpi and fixed in 4% paraformaldehyde for 3 days at room temperature and cut in vertical position. Next, the nasal blocks and BF sections were dehydrated in ascending serial grades of ethyl alcohol cleared in xylene and embedded in paraffin wax. The paraffin sections of both tissues were cut into 4 mm thick cross-sections using a microtome (LEICA RM 2015). The sections were then mounted on slides, deparaffinized in xylene, rehydrated by passing through descending serial grades of ethyl alcohol and stained with hematoxylin–eosin, mounted with neutral balsam. Finally, the stained sections were observed using Olympus BX51&DP 70 Digital Camera System.

### Immunohistochemistry and immunofluorescence staining

The fixed paraffin sections of bursa and nasal were cut into 4 mm sections. The IBDV positive cells were detected by immunohistochemistry (IHC) using primary mouse anti-VP2 polyclonal antibody, and the subsequent detection was completed using an HRP conjugated anti-mouse/anti-rabbit IgG SABC kit. The procedure for the control sample was the same as described above, except the primary antibodies were replaced with PBS. The quantification of VP2 positive cells was statistically determined at 40× magnification after counting five fields of each tissue in each chicken.

The 4 mm paraffin sections of bursa were used for co-localization immunofluorescence double staining. The non-specific antibodies binding sites were blocked by bovine serum albumin at room temperature for 30 min. Without washing, bursal sections were stained with mouse anti-VP2 polyclonal antibody (1:1000) overnight at 4 °C. The next day, sections were incubated with PE-conjugated goat anti-mouse IgG at 37 °C for 30 min. Subsequently, the sections were incubated with mouse anti-chicken Bu1 FITC (1:200) at 37 °C for 60 min. Finally, the sections were mounted with glycerin buffer, and examined under fluorescence microscope (SM-33TCI) immediately. The populations of IBDV-VP2 positive cells, Bu1 positive cells and double-positive cells were counted in five fields of each bursa at 20× magnification.

### Cell viability and plaque assay

The IBDV viral titer was also measured on DF1 cells by the plaque assay. Briefly, cells were grown in a 12-well plate and inoculated with undiluted or tenfold serially diluted cell supernatant. Then, we put 0.1 mL of pure or diluted virus (ranged from highest (10^–1^) to lowest (10^–5^) dilutions) into each well. After 1 h incubation, liquid was aspirated and covered with medium (DMEM with 2% FBS and 1% methylcellulose) at 37 °C for 3–5 days. After that, medium was removed and cells washed three times, then fixed with 4% PFA. Finally, plaques were stained with crystal violet and then washed with slow running water. The viral titer was calculated based on the method previously described by Reed and Muench [59].

### Bursal single-cell isolation and PBMC isolation

The BF from control and IBDV infected chicks at 12, 24, 36, 48 and 72 hpi were collected for flow cytometry and qRT-PCR analysis. To obtain a single-cell suspension, the BF was first minced using a sterile instrument and then filtered through a 70-μm nylon cell strainer (BD Falcon, San Jose, CA, USA). Subsequently, bursal cells were suspended in 5 mL medium of RPMI-1640 media supplemented with 5% heat-inactivated FBS and centrifuged at 300–400×*g* for 5 min at 4 °C. The pellet was incubated for 3 min at room temperature with 1 mL per chicken of ACK (Ammonium-Chloride-Potassium) lysing buffer to remove red blood cells in samples and centrifuged at 300–400×*g* for 5 min at 4 °C. The pellet was washed and suspended with the same medium and filtered with a 70-μm strainer. Finally, bursal cells were isolated for further use.

The blood was collected from control, and IBDV infected chicks at 1, 6 and 24 hpi in sterile heparinized syringes and was transferred to heparin tubes. Briefly, to isolate Peripheral blood mononuclear cells (PBMC), the whole blood was layered onto the Histopaque-1077 (Sigma-Aldrich, St. Louis, USA) in equal volume and centrifuged at 400×*g* for 30 min at room temperature. The upper plasma layer was removed, and the opaque interface layer containing white blood cells was carefully separated into another tube, washed twice with 5 mL PBS and centrifuged at 250×*g* for 10 min at room temperature. PBMC fixation was performed as previously explained by Lal et al. [60]. Briefly, the pellet was suspended in 1 mL of 1% paraformaldehyde to fix the cells, incubated for 30 min at room temperature, and centrifuged at 400×*g* for 5 min. The cells were washed twice with PBS and centrifuged at 400×*g* for 5 min. Finally, the pellet obtained was suspended in PBS with 1% bovine serum albumin. Cells were counted and equally divided into separate tubes for each treatment.

### Cell purification with magnetic beads

Bursal cells suspension isolated from bursa were counted and distributed equally (1 × 10^7^) into four tubes. The cells in separate tubes were stained with mouse anti-chicken Bu-1, mouse anti-chicken IgM, and mouse anti-chicken IgY antibody. After washing with MACS buffer, the cells were incubated with anti-mouse IgG microbeads anti-FITC for Bu1, anti-PE for IgM and anti-APC for IgY (Miltenyi Biotec) for 10 min at 4 °C in the dark and centrifuged at 300×*g* for 10 min. Cells were washed to remove the unbound antibodies with 1 mL MACS buffer and centrifuged as above. Then, the cell suspension was separated on MACS MS column on the magnetic field of a MACS Separator (Miltenyi Biotec) according to manufacturer’s instruction. The magnetic fraction of positively selected cells for each sample was further used in mRNA experiments.

### Flow cytometric analysis

The PBMC and bursa cell suspension from the bursa were collected from different treatments. For PBMC, the cells were equally separated about 1 × 10^6^, while bursal cells were separated into 1 × 10^7^ with a cell counter for each staining sample. To separate the whole hematopoietic cell or leukocytes in BF, the bursal cells were stained with mouse anti-chicken CD45. For B cells and T cells separation, the cells were stained with mouse anti-chicken Bu1 (FITC) and mouse anti-chicken CD4 (PE), respectively. In addition, to examine the immunoglobulins in B cells, PBMC and bursal cells were triple stained with Bu1, IgM and IgY. Followed by primary antibody staining, cells were kept for 30 min at 4 °C in the dark, according to the manufacturer’s instructions. After that, the cells were washed with chilled PBS, and the expression of surface markers was analyzed by FACS (BD FACS Calibur). The flow cytometry data were analyzed by FlowJo V10 software (Tree Star, San Carlos, CA, USA).

### Quantitative RT-PCR evaluation

Total RNA from different bursal single cell suspension, whole blood and PBMC, and DF1 cells of control and IBDV infected samples was extracted using TRIzol reagent (Invitrogen) according to the manufacturer’s protocol. Total RNA was used to synthesized complementary DNA (cDNA) by reverse transcription using HiScript TM qRT SuperMix (Vazyme) according to the manufacturer’s protocol. Subsequently, qRT-PCR was performed with SYBR Green qPCR Kit (TaKaRa) by the Applied Biosystems 7500 Fast Real-Time PCR System (Life Technologies) as previously described [61]. The gene expression level was normalized according to glyceraldehyde 3-phosphate dehydrogenase (*GAPDH*) expression. Control samples with no treatment were used as a standard for the quantitative analysis. All of the amplification primers used are listed in Table 1. All experiments were performed in triplicate, and relative expression levels were calculated using the 2−∆∆Ct method [62].

**Table 1:** qRT-PCR primers

Gene	Sense	Anti-sense
*GAPDH*	CACACAGAAGACGGTGGATG	AACAGAGACATTGGGGGTTG
*IBDV-A*	GCAATTGGGGAAGGTGTAGA	GTACCCCAGGTGAAGCAAGA
*IBDV-B*	ATATTGGCTCCCGAAGAACC	CAATGTTCATGGCAAAGGTG
*TLR3*	GCTATTGAGCAAAGTCGAGA	ACTCAGCGCACTTTACTATT
*MDA5*	GAACGAAAACCTGGGACAGA	AACAGCCACTCTGGTTTTGC
*LGP2*	AGTACGACCTGTGCCAGGAG	CGTCTTGCGACAAAACCTCT
*MAVS*	GAGGAAGACGTGGAGCTCAG	CTGTGGACTGTGCTTCTCCA
*STING*	TTCGTGGTGCTGTTCCTGT	AACACAGCACCTGCCAGAG
*TBK1*	AACGGACCAATTGAGTGGAG	GCAAGGTCATCTGCTGGAGT
*IRF7*	TCCCCAATGACAGAGGTTCT	GAGGGCAGAGATGTTGCAGT
*IFN-α*	GGACATGGCTCCCACACTAC	GGCTGCTGAGGATTTTGAAGA
*IFN-β*	CACCACCACCTTCTCCT	TGTGCGGTCAATCCAGT
*IFN-γ*	AACAACCTTCCTGATGGCGT	TGAAGAGTTCATTCGCGGCT
*IFNGR1*	TGGAGTCGGCAGAAGATGTT	GAAACCACTGGACCTGAGGA
*Dicer*	CGAGCTGTGTTGGTTGTTCTGG	AGGTCAAGTGAGGCAGGTGAGA
*Ago*	TCTGTAGGCAGTGGCTGTCAAC	GGAGCACAACACATCCAACACA
*Exportin5*	GGTACTCTCTTGCCCTCCAG	CTGGAAGCACCTGTTTGAGC

### Interferon treatment, enzyme-linked immunosorbent assay (ELISA) and Western blotting analysis

DF1 cells were pretreated with chIFN-ϒ for 12 h followed by IBDV infection 1000 IU/mL of interferons. The mock DF1 and interferon treated cultures were collected at the indicated time (12hpi and 24hpi) after IBDV infection. The supernatant was obtained from DF1 cells of mock and polyinosinic:polycytidylic acid (Poly I:C) or IBDV treated cells to perform ELISA of interleukins. Concentrations of IFN-α and IFN-ϒ in the supernatants were measured with ELISA kits (Shanghai Yili Biotechnology Co., Ltd. Shanghai, China) according to the manufacturer’s instructions. The sensitivity of the assay was 0.1 pg/mL for both IFN-α and IFN-ϒ. Total protein was isolated from DF1 cells treated differently according to the required experimentation indicated. The method was explained in our previous study [63].

### Single cell sequencing samples preparation

Chickens were divided into four different groups: S1—Control 2-week-old-chicks, S2—IBDV infected 2-week-old-chicks, S3—Control 3-week-old-chicks and S4—IBDV infected-3-week-old chicks. The whole bursa was collected and stored in MACS tissue storage solution (Miltenyi Biotec, Auburn, USA) to allow an optimized condition to the fresh organs, and transported directly to the research facility. Subsequently, each sample was minced to less than 1 mm cubic pieces on ice, followed by enzymatic digestion of TrypLE Express (Gibco) continuously shaking after every 5 min. Samples were then centrifuged at 300×*g* for 30 s at room temperature and and the supernatant carefully removed. Next, 1 × PBS (calcium and magnesium-free) containing 0.04% weight/volume BSA (400 µg/mL) was added and centrifugated at 300×*g* for 5 min at room temperature. The cell pellet was suspended in 1 mL red blood cell lysis buffer and incubated for 10 min at 4 °C. The samples were then resuspended in 1 mL PBS containing 0.04% BSA and filtered over Scienceware Flowmi 40-µm cell strainers (VMR). Finally, after sample dissociation, the cell concentration and cell viability were observed by hemocytometer and Trypan Blue staining method. Single-cell suspensions were loaded onto the Chromium Controller (10× Genomics) for droplet formation. Single-cell sequencing libraries were prepared using the Chromium Single Cell 3′Reagent Kit (10× Genomics) according to the manufacturer’s protocol. Samples were sequenced on the Illumina Novaseq6000 with 28-bp read,8-bp i7 index, and 98-bp read 2 for the gene-expression library.

### Single-cell RNA-seq data preprocessing

The accession number for the single cell sequence data reported in this paper is GEO GSE167377. The Cell Ranger software pipeline (version 3.1.0) provided by 10× Genomics was used to demultiplex cellular barcodes, map reads to the genome and transcriptome using the STAR aligner, and down-sample reads required to generate normalized aggregate data across samples, producing a matrix of gene counts versus cells. We processed the unique molecular identifier (UMI) count matrix using the R package Seurat [64] (version 3.1.1). To remove low-quality cells and likely multiple captures, which is a major concern in microdroplet-based experiments, we applied a criteria to filter out cells with UMI/gene numbers out of the limit of mean value ± twofold of standard deviations assuming a Guassian distribution of each cells' UMI/gene numbers. Following visual inspection of the distribution of cells by the fraction of mitochondrial genes expressed, only the mitochondrial UMIs across cells was four times than the median number cells were selected for subsequent analysis. After applying these QC criteria, single cells were included in downstream analyses. Library size normalization was performed with the NormalizeData function in Seurat to obtain the normalized count. Specifically, the global-scaling normalization method “LogNormalize” normalized the gene expression measurements for each cell by the total expression, multiplied by a scaling factor (10,000 by default), and the results were log-transformed.

Top variable genes across single cells were identified using the method described in Macosko et al. [65] The most variable genes were selected using FindVariableGenes function (mean.function = "FastExpMean",dispersion.function = "FastLogVMR") in Seurat [64]. Principal component analysis (PCA) was performed to reduce the dimensionality with the RunPCA function in Seurat [64]. Graph-based clustering was performed to cluster cells according to their gene expression profile using the FindClusters function in Seurat [64]. Cells were visualized using a 2-dimensional t-distributed stochastic neighbour embedding (t-SNE) algorithm with the RunTSNE function in Seurat [64]. We used the FindAllMarkers function (test.use = bimod) in Seurat [64] to identify marker genes of each cluster. For a given cluster, FindAllMarkers identified positive markers compared with all other cells. Then, we used the R package SingleR, a novel computational method for unbiased cell type recognition of scRNA-seq, with the reference transcriptomic datasets ‘mouse Primary Cell Atlas’ (Immgen: The Immunological Genome Project: networks of gene expression in immune cells) to infer the cell of origin of each of the single cells independently and identify cell types. Differentially expressed genes (DEGs) were identified using the FindMarkers function (test.use = MAST) in Seurat [64]. p value < 0.05 and |log2foldchange| > 0.58 was set as the threshold for significantly differential expression.

### Statistical analysis

Results were expressed as the means ± SD and analyzed with GraphPad Prism v6.01 (San Diego, US) or SPSS 17.0. One-way analysis of variance (ANOVA) was employed to determine significant differences among multiple groups, followed by Tukey’s or Dunnett’s multiple comparison tests. Differences were considered to be statistically significant when p < 0.05. Statistical significance in the figures is indicated as follows: ****p < 0.0001, ***p < 0.001, **p < 0.01, *p < 0.05; ns, not significant. Data were combined from at least three independent experiments unless otherwise stated.

## Supplementary Information

**Additional file 1: Figure S1. Microscopical lesions in bursa of Fabricious (BF) and single-cells identified five major cells in each sample shown in the graph. A**: Microscopical lesions in bursa of Fabricious (BF) following intranasal inoculation of IBDV strain BC6/85. Paraffin sections of bursa from two weeks and three weeks old SPF control and IBDV infected chickens (left controls and right infected; 40 ×) were examine for histopathological changes through HE staining. At 72hpi, the follicular lymphoid apoptosis and depletion in lymphoid follicles were observed in bursa. Control represents the uninfected control groups. **B**: Total cell population of the major five cells in each sample shown in graph. Green dot represents blank of two-weeks-old-chicks bursa, red dot represents IBDV infected two-weeks-old-chicks bursa cells, blue dot show blank of three-weeks-old-chicks bursa, and brown dot represents IBDV infected three-weeks-old-chicks bursal cells population in each of the five major immune and non-immune cell types.

**Additional file 2: Figure S2. Single-cell analysis of infected and non-infected age-dependent bursal samples and host reveals cell prevalence of viral genome in bursal derived cell types. A**: Analysis of whole cells into different t-SNE sample distribution. Samples were collected from four differently treated chicken’s bursa, i.e., blank two-weeks-old-chicks bursa (light brow), IBDV infected two-weeks-old-chicks bursa (dark brown), blank three-weeks-old-chicks bursa (grey), IBDV infected three-weeks-old-chicks bursa (light blue). **B**: Sample population analyzed into nine major clusters shown in different colours for each sample in separate t-SNE distribution, **C**: and bar graph distribution. **D**: Single-cell heterogeneity of intracellular viral load of two segments of IBDV strain BC6/85 (BC6/85-A and BC6/85-B) within the IBDV treated two-weeks and three-weeks-old-chicks bursa shown in t-SNE distribution. **E**: Intracellular viral load of IBDV strain BC6/85-A and BC6/85-B within the IBDV treated hosts shown in the nine major clusters of cells distributed through violin plot.

**Additional file 3: Figure S3. B cell Class Switching and migration analysis. A**: t-SNE and violin plot presentation of *BCL6, PAX5* (left) and *IRF4, BLIMP1* (right) in B cell population. **B**: t-SNE and violin plot show *CXCR4, CXCL12* (left) and, *CXCR5, CXCL13* (right) in B cell population. Violin plot shows the transcription level of each gene in each sample type.

**Additional file 4: Figure S4. Dendritic cells distribution and characterization in bursa. A**: UMAP visualization of the five major cells types in bursal population shown in unique clusters and different color distribution. The encircled grey cells clusters indicate the total dendritic cells population. **B**: UMAP graphical presentation of the dendritic cells into four different sub-population based on the mRNA transcriptional profiling, shown in different colors. **C**: UMAP representation of dendritic cells into macrophages (macrophage-1 in red and macrophage-2 in violet) and dendritic cells subtypes (conventional dendritic cells “cDC”, plasmoid dendritic cells “pDC”, and monocytes and dendritic cells “Mo-DC”) based on the differential gene expression encircled. **D**: UMAP graph of four dendritic cells clusters into normal and virus-infected hosts samples shown in different color distribution. **E**: Heatmap of the mRNA expression profiling in dendritic cells population distributed based on dendritic cells clusters difference, shown at the bottom of the heatmap. **F**: UMAP visualization of the viral load (shown in colored plus sign) of IBDV strain BC6/85-A and BC6/85-B in IBDV infected groups.

**Additional file 5: Figure S5. UMAP and violin plot of dendritic cells subtypes**. **A**: Langerhans dendritic cells (LDC) blue, **B**: bursal secretory dendritic cells (BSDC) brown, **C**: and follicular dendritic cells (FDC) in violet; distribution in dendritic cells population (left), and violin plot show cell fraction of dendritic cells subtypes (LDC, BSDC and FDC) in each sample.

**Additional file 6: Figure S6. The expression of chicken RNA sensing pathway after IBDV infection in bursa. A**: Results of qPCR analysis following stimulation by Poly I:C and IBDV at 12hpi and 24hpi of RNA sensing pathway-related genes: *TLR3, MDA5, LGP2*, and *MAVS*. **B**: Pie chart showing the percentage of *TLR3/MDA5/LGP2/MAVS* combined transcriptome level in each cell type. **C**: Graph figure showing the combined gene transcriptome level of *TLR3/MDA5/LGP2/MAVS* in control and infected groups in each cell type. **D**: t-SNE (left side) and violin plot (right side) of *TLR3/MDA5/LGP2/MAVS* in each cell population. Violin plots show expression in each (control and infected) sample type. The level of significance between blank and treated groups are identified by * p < 0.05, ** p < 0.01, *** p < 0.001, and **** p < 0.0001, determined by one-way ANOVA with Tukey’s multiple comparison test.

**Additional file 7: Figure S7. The expression of chicken *STING/TBK1/IRF7* pathway after IBDV infection in bursa. A:** Results of qPCR analysis following stimulation by Poly I:C and IBDV at 12hpi and 24hpi of *STING, TBK1*, and *IRF7*. **B:** Pie chart showing the percentage of *STING/TBK1/IRF7* combined transcriptome level in each cell type. **C:** Graphical figure showing the combined gene transcriptome level of *STING/TBK1/IRF7* in control and infected groups in each cell type. **D:** t-SNE (left side) and violin plot (right side) of *STING/TBK1/IRF7* in each cell population. Violin plots show expression in each (control and infected) sample type. The level of significance between blank and treated groups are identified by * p < 0.05, ** p < 0.01, *** p < 0.001, and **** p < 0.0001, determined by one-way ANOVA with Tukey’s multiple comparison test.

**Additional file 8: Table S1. Key Resources Tables.**

**Additional file 9: Table S2. DEGs of Genes Unique to Each Cell Type.**

**Additional file 10.** Excel data of total 42,484 single transcriptomes were distributed into nine different clusters based on the differential expression levels.

**Additional file 11.** Excel data of total single transcriptomes divided into five clusters based on cell markers.

**Additional file 12.** Excel data of B cells were segregated into five different clusters based on differential surface marker expression.

**Additional file 13.** Excel data of epithelial cells were divided into nine subpopulations based on differential surface marker expression.

**Additional file 14.** Excel data of DC population were divided into four distinct clusters based on differential surface marker expression.

## Data Availability

Additional Data are now available at NAR online. The accession number for the single cell sequence data reported in this paper is GEO GSE167377.
