# Supplementary figures and images for: From nasal to basal: single-cell sequencing of the bursa of Fabricius highlights the IBDV infection mechanism in chickens
Source: Cell Biosci. 2021 Dec 16;11:212. doi: 10.1186/s13578-021-00728-9 (PMC8675306; doi:10.1186/s13578-021-00728-9)

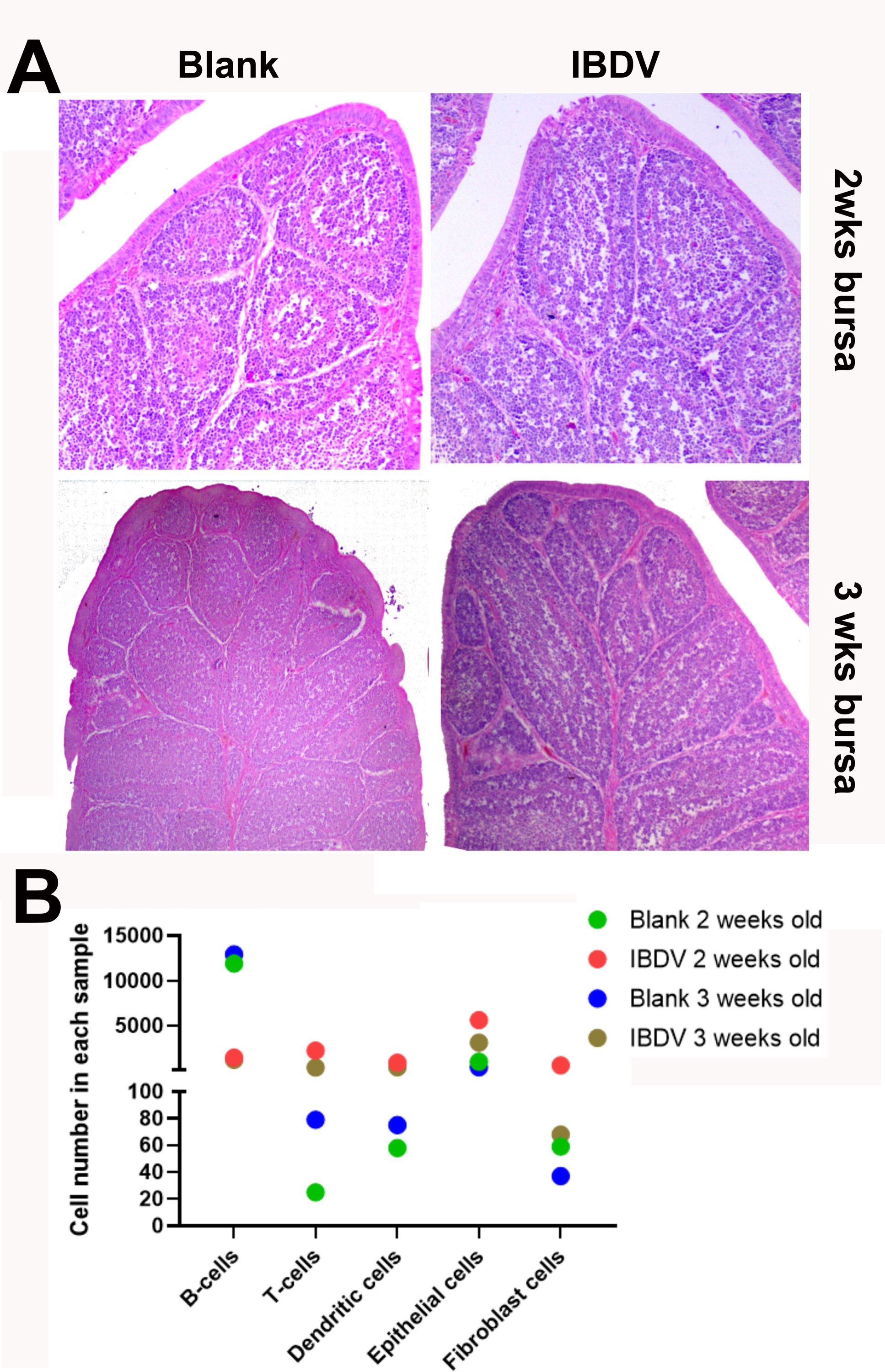

Supplement: Supplementary file 1 — Additional file 1: Figure S1. Microscopical lesions in bursa of Fabricious (BF) and single-cells identified five major cells in each sample shown in the graph. A: Microscopical lesions in bursa of Fabricious (BF) following intranasal inoculation of IBDV strain BC6/85. Paraffin sections of bursa from two weeks and three weeks old SPF control and IBDV infected chickens (left controls and right infected; 40 ×) were examine for histopathological changes through HE staining. At 72hpi, the follicular lymphoid apoptosis and depletion in lymphoid follicles were observed in bursa. Control represents the uninfected control groups. B: Total cell population of the major five cells in each sample shown in graph. Green dot represents blank of two-weeks-old-chicks bursa, red dot represents IBDV infected two-weeks-old-chicks bursa cells, blue dot show blank of three-weeks-old-chicks bursa, and brown dot represents IBDV infected three-weeks-old-chicks bursal cells population in each of the five major immune and non-immune cell types. [file 13578_2021_728_MOESM1_ESM.tif]

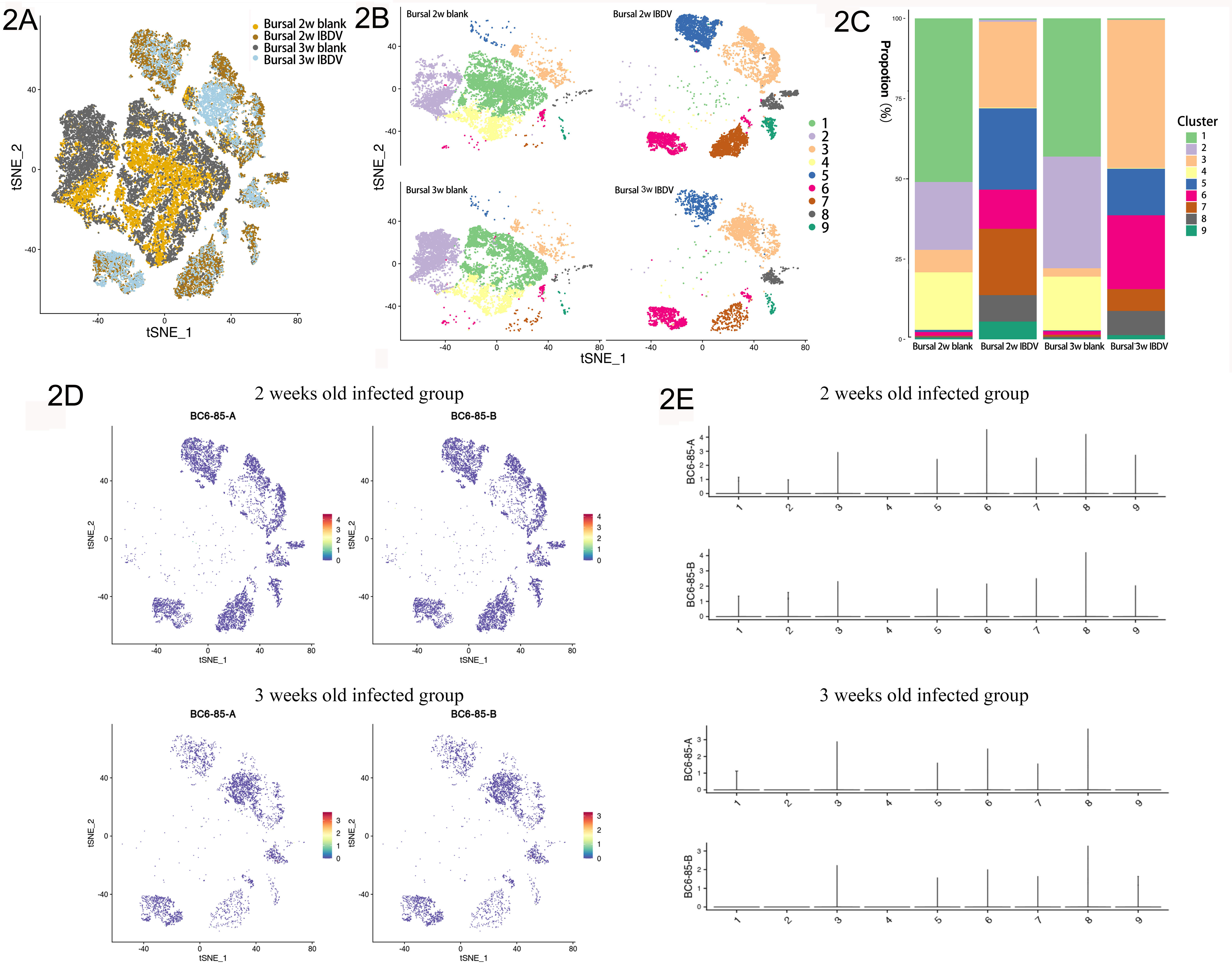

Supplement: Supplementary file 2 — Additional file 2: Figure S2. Single-cell analysis of infected and non-infected age-dependent bursal samples and host reveals cell prevalence of viral genome in bursal derived cell types. A: Analysis of whole cells into different t-SNE sample distribution. Samples were collected from four differently treated chicken’s bursa, i.e., blank two-weeks-old-chicks bursa (light brow), IBDV infected two-weeks-old-chicks bursa (dark brown), blank three-weeks-old-chicks bursa (grey), IBDV infected three-weeks-old-chicks bursa (light blue). B: Sample population analyzed into nine major clusters shown in different colours for each sample in separate t-SNE distribution, C: and bar graph distribution. D: Single-cell heterogeneity of intracellular viral load of two segments of IBDV strain BC6/85 (BC6/85-A and BC6/85-B) within the IBDV treated two-weeks and three-weeks-old-chicks bursa shown in t-SNE distribution. E: Intracellular viral load of IBDV strain BC6/85-A and BC6/85-B within the IBDV treated hosts shown in the nine major clusters of cells distributed through violin plot. [file 13578_2021_728_MOESM2_ESM.tif]

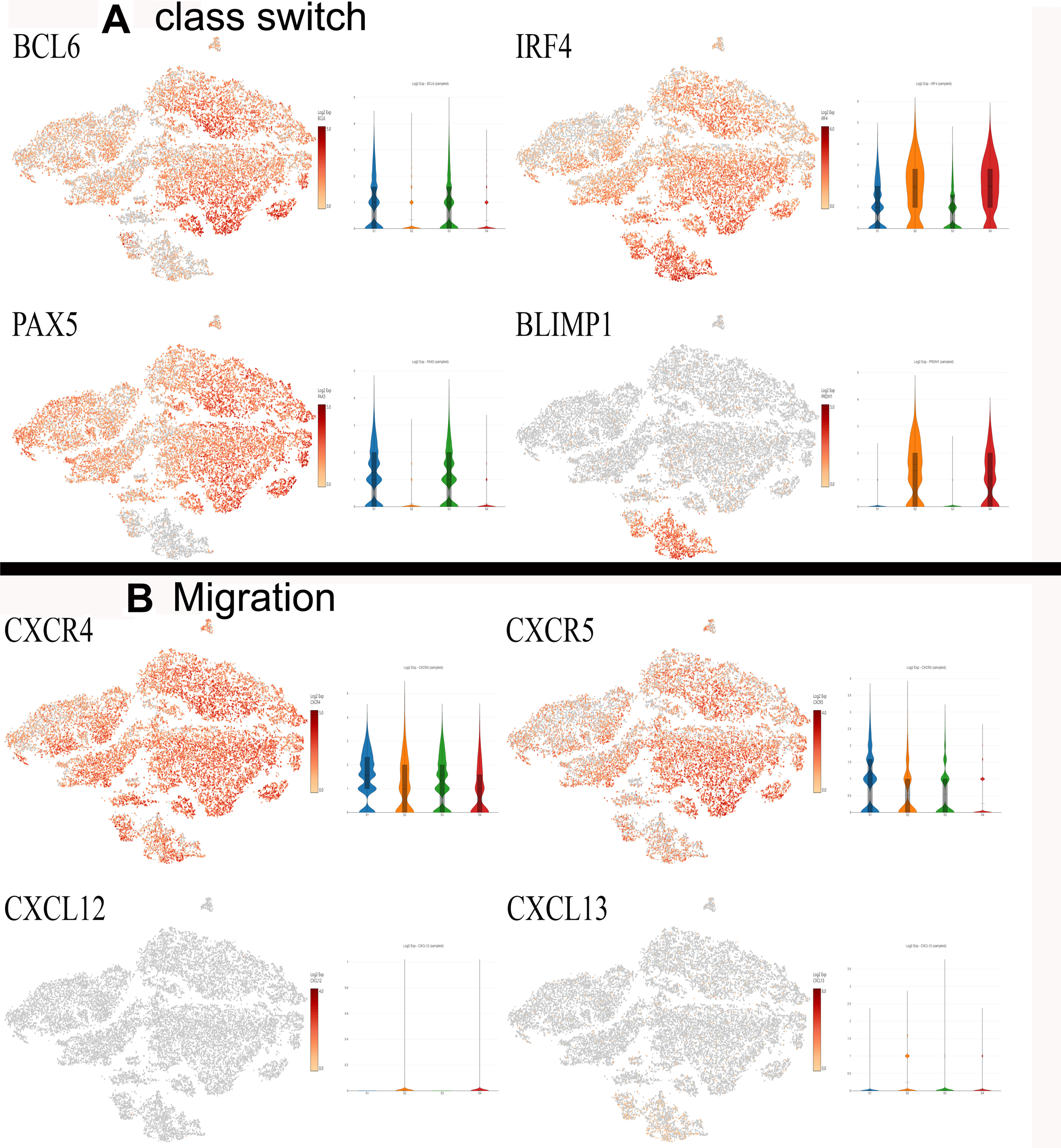

Supplement: Supplementary file 3 — Additional file 3: Figure S3. B cell Class Switching and migration analysis. A: t-SNE and violin plot presentation of BCL6, PAX5 (left) and IRF4, BLIMP1 (right) in B cell population. B: t-SNE and violin plot show CXCR4, CXCL12 (left) and, CXCR5, CXCL13 (right) in B cell population. Violin plot shows the transcription level of each gene in each sample type. [file 13578_2021_728_MOESM3_ESM.jpg]

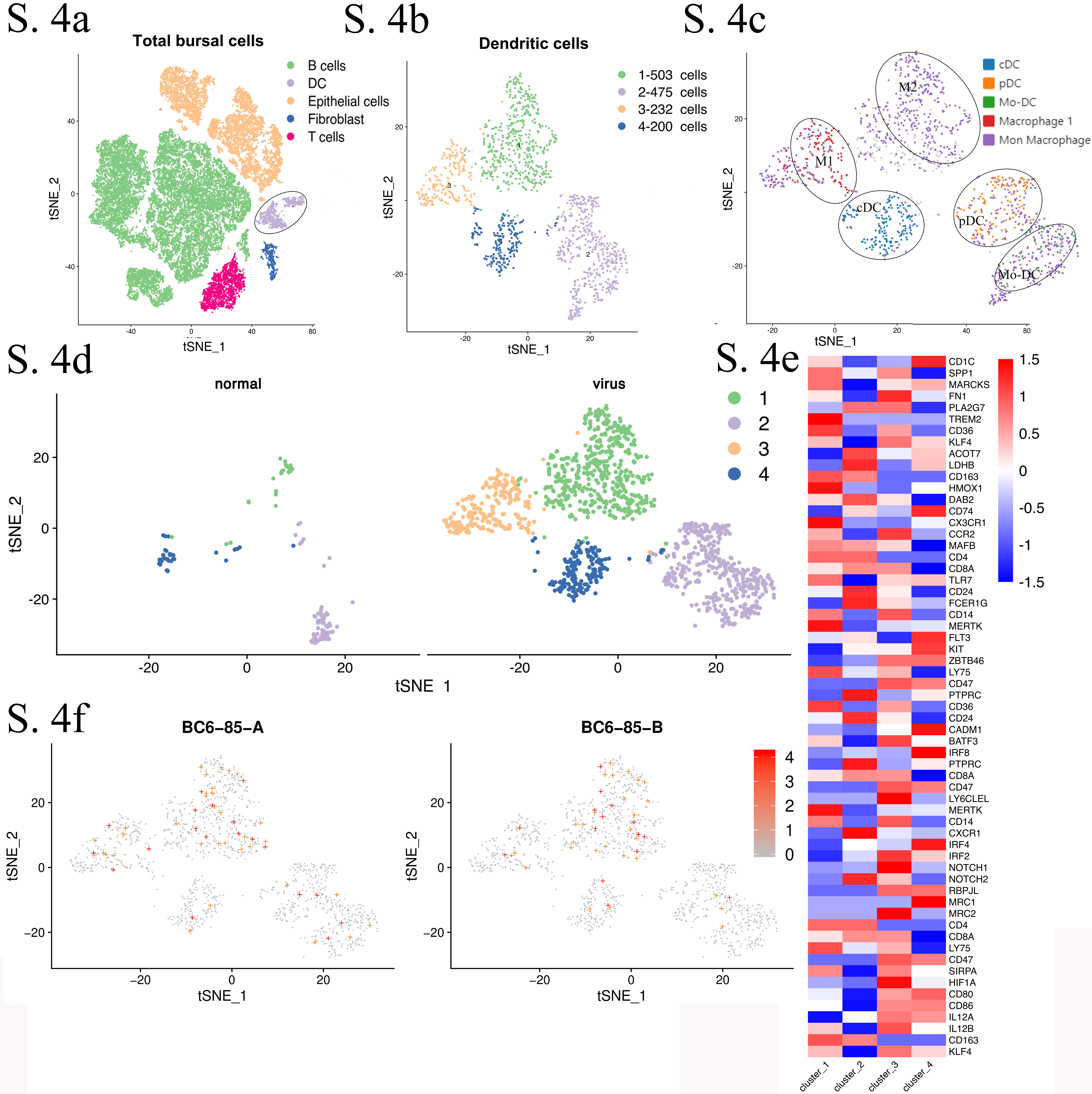

Supplement: Supplementary file 4 — Additional file 4: Figure S4. Dendritic cells distribution and characterization in bursa. A: UMAP visualization of the five major cells types in bursal population shown in unique clusters and different color distribution. The encircled grey cells clusters indicate the total dendritic cells population. B: UMAP graphical presentation of the dendritic cells into four different sub-population based on the mRNA transcriptional profiling, shown in different colors. C: UMAP representation of dendritic cells into macrophages (macrophage-1 in red and macrophage-2 in violet) and dendritic cells subtypes (conventional dendritic cells “cDC”, plasmoid dendritic cells “pDC”, and monocytes and dendritic cells “Mo-DC”) based on the differential gene expression encircled. D: UMAP graph of four dendritic cells clusters into normal and virus-infected hosts samples shown in different color distribution. E: Heatmap of the mRNA expression profiling in dendritic cells population distributed based on dendritic cells clusters difference, shown at the bottom of the heatmap. F: UMAP visualization of the viral load (shown in colored plus sign) of IBDV strain BC6/85-A and BC6/85-B in IBDV infected groups. [file 13578_2021_728_MOESM4_ESM.tif]

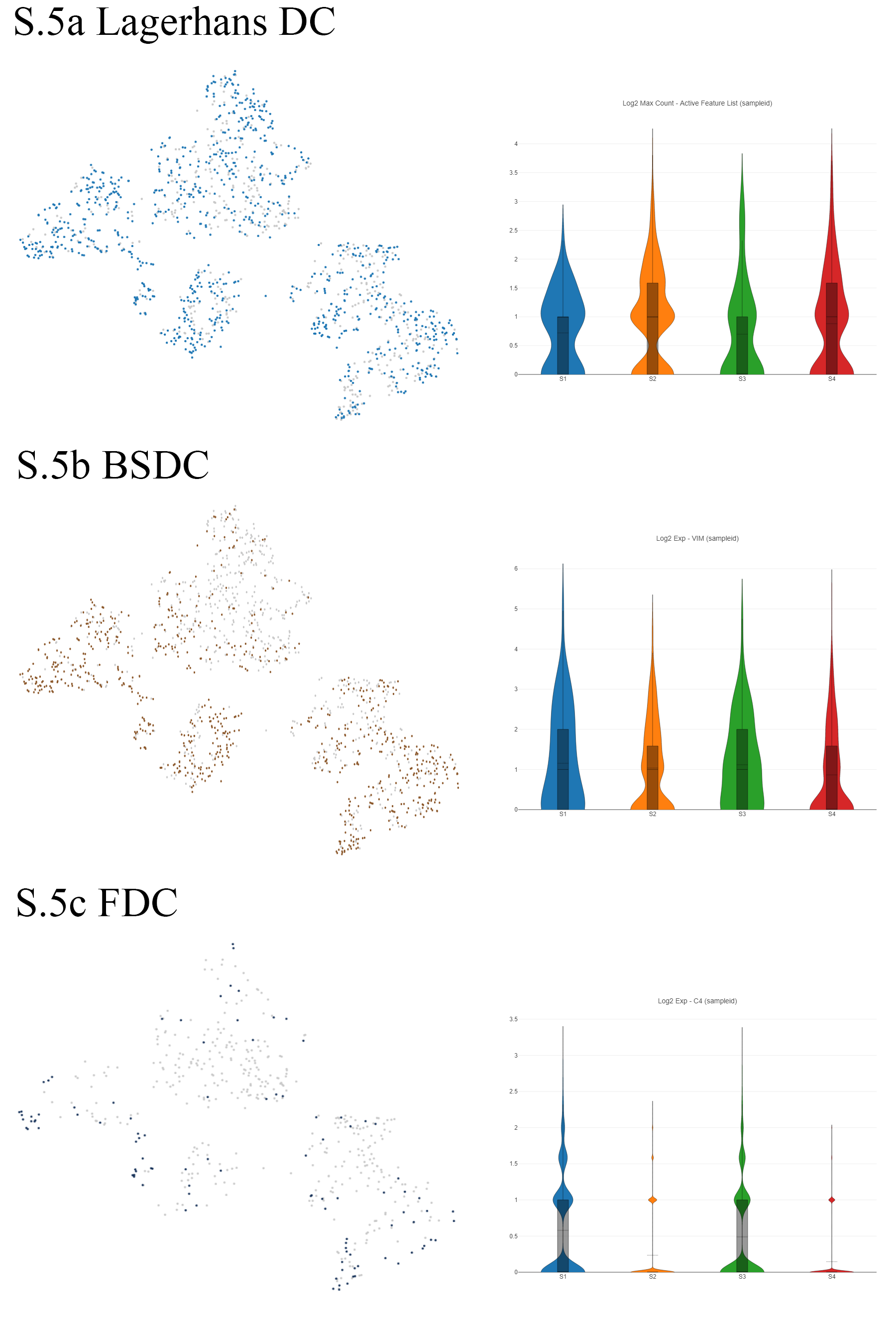

Supplement: Supplementary file 5 — Additional file 5: Figure S5. UMAP and violin plot of dendritic cells subtypes. A: Langerhans dendritic cells (LDC) blue, B: bursal secretory dendritic cells (BSDC) brown, C: and follicular dendritic cells (FDC) in violet; distribution in dendritic cells population (left), and violin plot show cell fraction of dendritic cells subtypes (LDC, BSDC and FDC) in each sample. [file 13578_2021_728_MOESM5_ESM.tif]

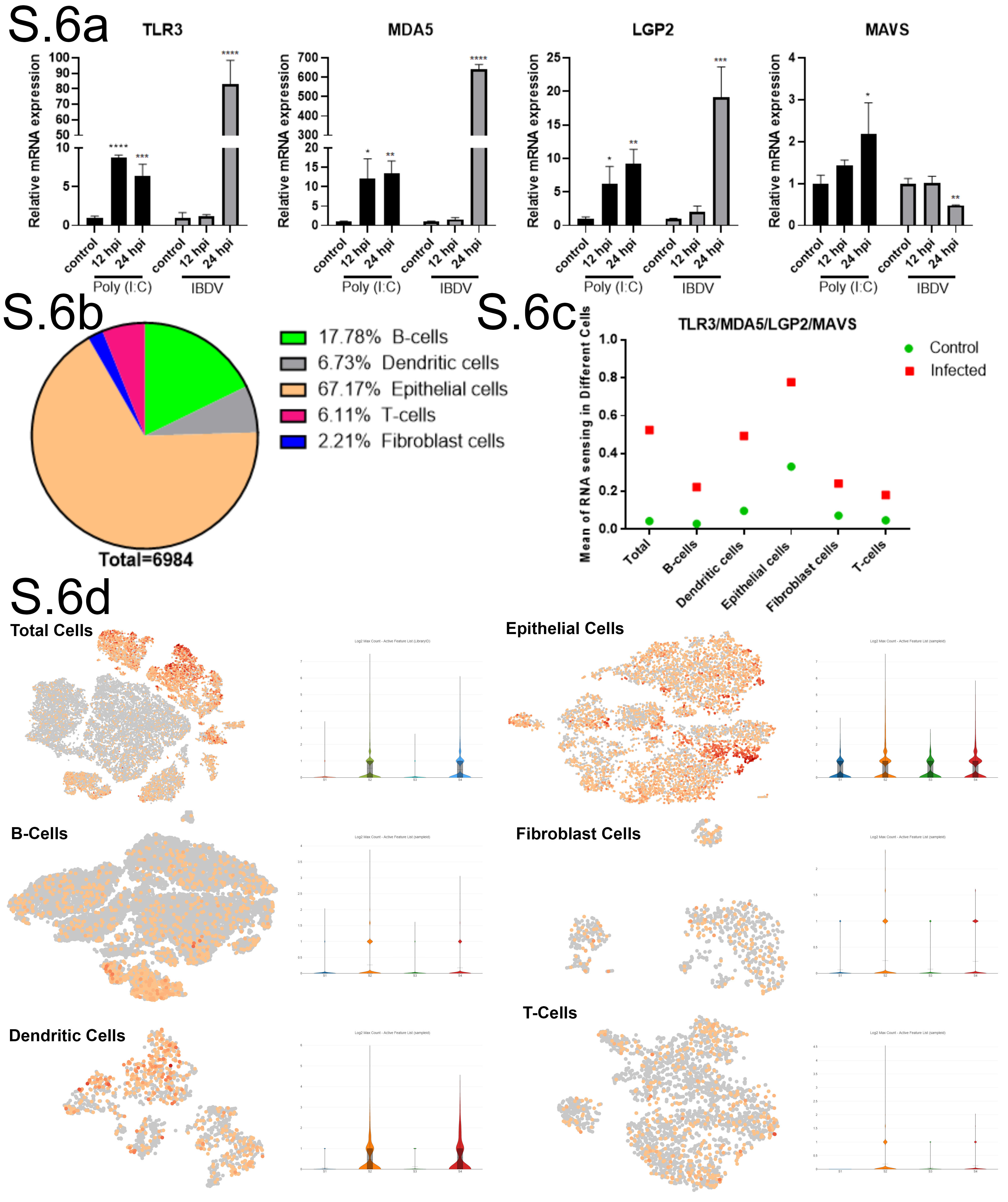

Supplement: Supplementary file 6 — Additional file 6: Figure S6. The expression of chicken RNA sensing pathway after IBDV infection in bursa. A: Results of qPCR analysis following stimulation by Poly I:C and IBDV at 12hpi and 24hpi of RNA sensing pathway-related genes: TLR3, MDA5, LGP2, and MAVS. B: Pie chart showing the percentage of TLR3/MDA5/LGP2/MAVS combined transcriptome level in each cell type. C: Graph figure showing the combined gene transcriptome level of TLR3/MDA5/LGP2/MAVS in control and infected groups in each cell type. D: t-SNE (left side) and violin plot (right side) of TLR3/MDA5/LGP2/MAVS in each cell population. Violin plots show expression in each (control and infected) sample type. The level of significance between blank and treated groups are identified by * p < 0.05, ** p < 0.01, *** p < 0.001, and **** p < 0.0001, determined by one-way ANOVA with Tukey’s multiple comparison test. [file 13578_2021_728_MOESM6_ESM.tif]

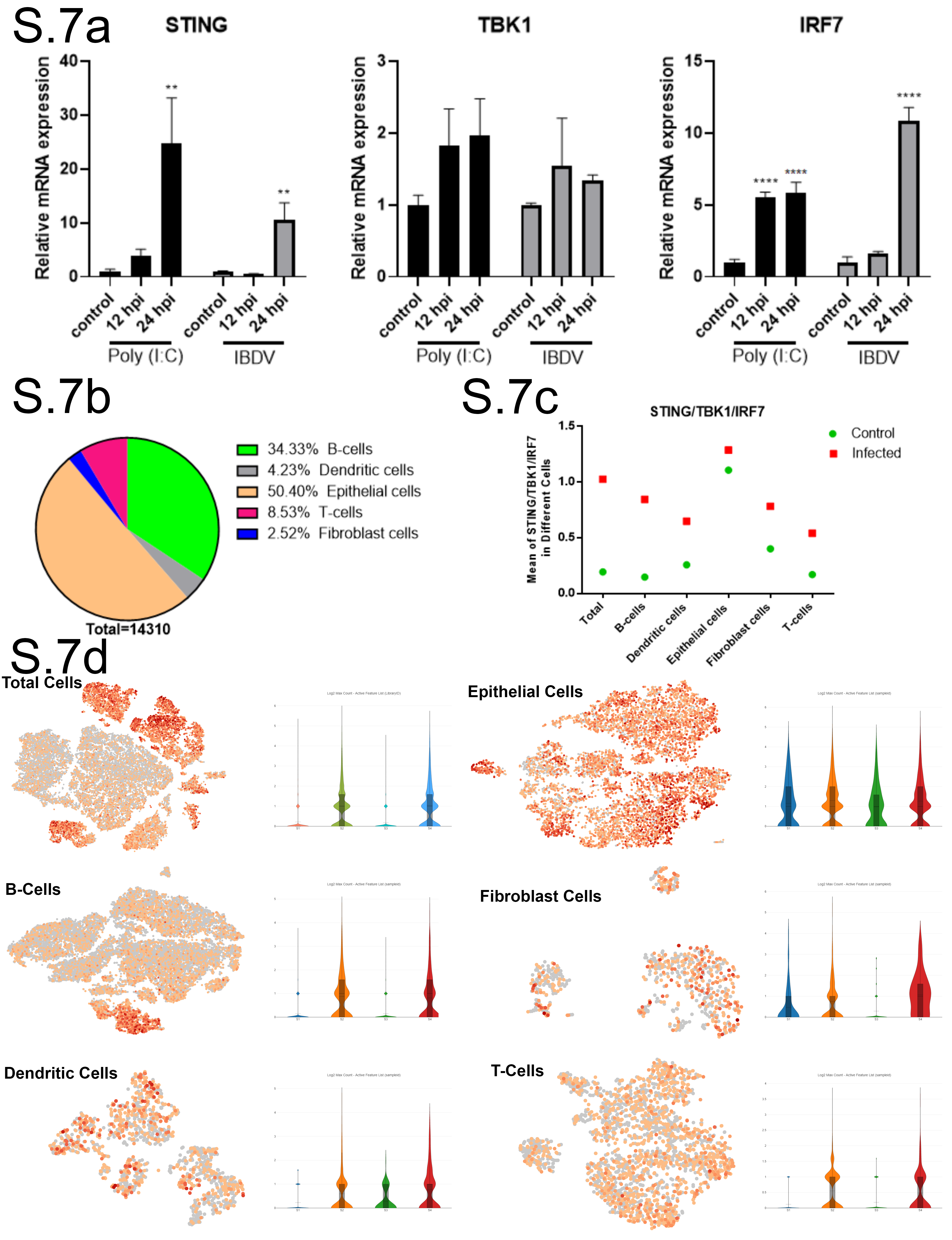

Supplement: Supplementary file 7 — Additional file 7: Figure S7. The expression of chicken STING/TBK1/IRF7 pathway after IBDV infection in bursa. A: Results of qPCR analysis following stimulation by Poly I:C and IBDV at 12hpi and 24hpi of STING, TBK1, and IRF7. B: Pie chart showing the percentage of STING/TBK1/IRF7 combined transcriptome level in each cell type. C: Graphical figure showing the combined gene transcriptome level of STING/TBK1/IRF7 in control and infected groups in each cell type. D: t-SNE (left side) and violin plot (right side) of STING/TBK1/IRF7 in each cell population. Violin plots show expression in each (control and infected) sample type. The level of significance between blank and treated groups are identified by * p < 0.05, ** p < 0.01, *** p < 0.001, and **** p < 0.0001, determined by one-way ANOVA with Tukey’s multiple comparison test. [file 13578_2021_728_MOESM7_ESM.tif]
