# Supplementary material for: From nasal to basal: single-cell sequencing of the bursa of Fabricius highlights the IBDV infection mechanism in chickens
Source: Cell Biosci. 2021 Dec 16;11:212. doi: 10.1186/s13578-021-00728-9 (PMC8675306; doi:10.1186/s13578-021-00728-9)
Supplement: Supplementary file 8 — Additional file 8: Table S1. Key Resources Tables. [file 13578_2021_728_MOESM8_ESM.docx]

**Additional file 8: Table S1. Key Resources Tables**

| **Antibodies** |  |  |
| --- | --- | --- |
| Mouse anti-chicken Bu1-FITC | Southern Biotech | CAT No: 8395-02 |
| Mouse anti-chicken CD4-PE | Southern Biotech | CAT No: 8210-09 |
| Mouse anti-chicken CD45-APC | Southern Biotech | CAT No: 8270-11 |
| Mouse anti-chicken MHCII-PE | Southern Biotech | CAT No:8350-09 |
| Mouse anti-chicken IgM-PE | Southern Biotech | CAT No: 8310-09 |
| Mouse anti-chicken IgY-APC | Southern Biotech | CAT No: 8320-31 |
| Mouse anti-VP2 polyclonal antibody | N/A | N/A |
| Goat anti-mouse IgG (H+L) PE-conjugated | Multi Sciences | CAT No: GAM0041 |
| HRP conjugated anti-mouse/anti-rabbit IgG SABC kit | BOSTER | N/A |
| Hoechst 33342 (H3570) | Life Technologies | N/A |

| **Critical Commercial Assays** |  |  |
| --- | --- | --- |
| RPMI 1640 | GIBCO | N/A |
| Fetal Bovine Serum (FBS) | GIBCO | N/A |
| ACK lysis buffer | GIBCO | REF # A10492-01 |
| Histopaque-1077 | Sigma-Aldrich | Lot # RNBJ0383 |
| Mini MACS starting kit, MS columns, and assembly | Miltenyi Biotech | N/A |
| Anti-FITC Micro-Beads | Miltenyi Biotech | Mat # 120-008-613 |
| Anti-APC Micro-Beads | Miltenyi Biotech | Mat # 120-008-867 |
| Anti-PE Micro-Beads | Miltenyi Biotech | Mat # 120-008-614 |
| 70-μm nylon cell strainer | BD Falcon | N/A |
| TRIzol | Invitrogen | N/A |
| QuantiTect SYBR Green PCR master mix | TaKaRa | N/A |
| HiScript TM QRT SuperMix | Vazyme | N/A |
| FlowJo V10 software | FlowJo Software | N/A |
| GraphPad Prism v9 | GraphPad Software | N/A |
