# Supplementary material for: From nasal to basal: single-cell sequencing of the bursa of Fabricius highlights the IBDV infection mechanism in chickens
Source: Cell Biosci. 2021 Dec 16;11:212. doi: 10.1186/s13578-021-00728-9 (PMC8675306; doi:10.1186/s13578-021-00728-9)
Supplement: Supplementary file 9 — Additional file 9: Table S2. DEGs of Genes Unique to Each Cell Type. [file 13578_2021_728_MOESM9_ESM.docx]

**Additional file 9: Table S2. DEGs of Genes Unique to Each Cell Type**

| Cell Type | Gene |
| --- | --- |
| B cells | PAX5 |
|  | LOC396098 |
|  | LOC419333 |
|  | LOC112531844 |
|  | BLVRA |
|  | TNFRSF13C |
|  | SCGF |
|  | LOC100857302 |
|  | CD1C |
|  | WDR66 |
| Epithelial Cells | RHOV |
|  | SRAPL |
|  | SOX9 |
|  | KRT80 |
|  | PPL |
|  | ZNF750 |
|  | VILL |
|  | FOXA1 |
|  | CLDN4 |
|  | SPDEF |
| T cells | TCF7 |
|  | BCL11B |
|  | CNKSR2L |
|  | RORA |
|  | CTLA4 |
|  | UBASH3A |
|  | TOX2 |
|  | TRAT1 |
|  | MAF |
|  | ITK |
| Dendritic cells | INHBA |
|  | ARHGAP22 |
|  | RASGEF1B |
|  | DCLK3 |
|  | HTRA1 |
|  | CCL4 |
|  | CSF1R |
|  | FYB |
|  | FCER1G |
|  | DOCK4 |
| Fibroblast cells | COL5A1 |
|  | COL6A1 |
|  | DCN |
|  | COL6A2 |
|  | RARRES2 |
|  | THY1 |
|  | IGFBP4 |
|  | SDC2 |
|  | COL6A3 |
|  | LOXL1 |
